# The difference between shorter- versus longer-term psychotherapy for adult mental health disorders: a systematic review with meta-analysis

**DOI:** 10.1186/s12888-023-04895-6

**Published:** 2023-06-16

**Authors:** Sophie Juul, Janus Christian Jakobsen, Caroline Kamp Jørgensen, Stig Poulsen, Per Sørensen, Sebastian Simonsen

**Affiliations:** 1 Stolpegaard Psychotherapy Centre, Mental Health Services in the Capital Region of Denmark, Gentofte, Denmark; 2 Copenhagen Trial Unit, Centre for Clinical Intervention Research, Copenhagen University Hospital – Rigshospitalet, The Capital Region of Denmark, Copenhagen, Denmark; 3 Department of Regional Health Research, Faculty of Health Sciences, University of Southern Denmark, Odense, Denmark; 4 Department of Psychology, University of Copenhagen, Copenhagen, Denmark

**Keywords:** Mental health disorders, Duration of psychotherapy, Systematic review, Meta-analysis

## Abstract

**Background:**

The optimal psychotherapy duration for mental health disorders is unclear. Our aim was to assess the beneficial and harmful effects of shorter- versus longer-term psychotherapy for adult mental health disorders.

**Method:**

We searched relevant databases and websites for published and unpublished randomised clinical trials assessing different durations of the same psychotherapy type before June 27, 2022. Our methodology was based on Cochrane and an eight-step procedure. Primary outcomes were quality of life, serious adverse events, and symptom severity. Secondary outcomes were suicide or suicide-attempts, self-harm, and level of functioning.

**Results:**

We included 19 trials randomising 3,447 participants. All trials were at high risk of bias. Three single trials met the required information size needed to confirm or reject realistic intervention effects. One single trial showed no evidence of a difference between 6 versus 12 months dialectical behavioral therapy for borderline personality when assessing quality of life, symptom severity, and level of functioning. One single trial showed evidence of a beneficial effect of adding booster sessions to 8 and 12 weeks of internet-based cognitive behavioral therapy for depression and anxiety when assessing symptom severity and level of functioning. One single trial showed no evidence of a difference between 20 weeks versus 3 years of psychodynamic psychotherapy for mood- or anxiety disorders when assessing symptom severity and level of functioning. It was only possible to conduct two pre-planned meta-analyses. Meta-analysis showed no evidence of a difference between shorter- and longer-term cognitive behavioural therapy for anxiety disorders on anxiety symptoms at end of treatment (SMD: 0.08; 95% CI: -0.47 to 0.63; *p* = 0.77; I^2^ = 73%; four trials; very low certainty). Meta-analysis showed no evidence of a difference between shorter and longer-term psychodynamic psychotherapy for mood- and anxiety disorders on level of functioning (SMD 0.16; 95% CI -0.08 to 0.40; *p* = 0.20; I^2^ = 21%; two trials; very low certainty).

**Conclusions:**

The evidence for shorter versus longer-term psychotherapy for adult mental health disorders is currently unclear. We only identified 19 randomised clinical trials. More trials at low risk of bias and at low risk of random errors assessing participants at different levels of psychopathological severity are urgently needed.

**Systematic review registration:**

PROSPERO CRD42019128535.

**Supplementary Information:**

The online version contains supplementary material available at 10.1186/s12888-023-04895-6.

## Background

The annual prevalence of mental health disorders is estimated to be 38.2% of the European population [1]. The economic burden from mental health disorders is high, both because of direct health care costs, but also because of indirect costs like sick days, disability, and early retirement [1–3]. Psychotherapy is among the recommended and widely used interventions for most disorders [4]. Accordingly, it would be highly relevant to identify the optimal duration of psychotherapy for various mental health disorders and conditions. If short-term psychotherapy is the optimal treatment approach for a given disorder, this could result in a reduction of waitlists and thus a greater access to evidence-based care. On the contrary, if long-term psychotherapy is the most optimal treatment, it would be sensible for mental health systems to invest in these treatments, as they would translate into greater long-term health and occupational benefits [5, 6].

The relationship between dose and effect in psychotherapy has been studied with mixed results in non-controlled studies [5, 7]. While several non-controlled studies indicate that there is a linear or negatively accelerating relationship between number of psychotherapy sessions and outcome for most mental health disorders [8, 9], these findings have been criticized on methodological grounds [10].

The inconclusiveness of the existing research and the general lack of internal validity of non-controlled studies [11, 12] indicate the need for a systematic review of well-designed randomised clinical trials directly comparing psychotherapies of different durations for clearly specified populations, including patients treated for mental health disorders in secondary mental health care settings [11, 12]. However, such systematic review has not previously been performed [6].

The present systematic review aims at forming the basis for evidence-based guideline recommendations for the optimal duration of psychotherapy for adult mental health disorders taking both benefits and harms, bias risk (systematic errors), play of chance (random errors), and certainty of the findings into consideration.

## Methods

We report this systematic review in accordance with the Preferred Reporting Items for Systematic Reviews and Meta-Analysis (PRISMA) guidelines [13] A PRISMA 2020 checklist can be found in Supplementary material 1. The Cochrane methodology used in this systematic review is described in detail in our protocol [6], which was also registered in the PROSPERO database (CRD42019128535) prior to the systematic literature search.

### Search strategy and selection criteria

#### Electronic searches

An experienced information specialist searched for eligible trials comparing a shorter with a longer-term version of the same psychotherapy type for one or more adult mental health published before June 27, 2022 in the following databases: Cochrane Central Register of Controlled Trials (CENTRAL), Medical Literature Analysis and Retrieval System Online (MEDLINE), Excerpta Medica database (EMBASE), Latin American and Caribbean Health Sciences Literature (LILACS), PsycINFO, Science Citation Index Expanded (SCI-EXPANDED), Social Sciences Citation Index (SSCI), Conference Proceedings Citation Index—Science (CPCI-S), and Conference Proceedings Citation Index—Social Science & Humanities (CPCI-SSH). The electronic search strategies can be found in Supplementary material 2. Additionally, we checked the reference lists of relevant publications for any unidentified trials, and we hand searched conference abstracts from psychiatry conferences for relevant trials. We also considered unpublished and gray literature trials if these were identified.

#### Inclusion and exclusion criteria

We only included randomized clinical trials. Trials were included irrespective of setting, publication status, publication year, language, and the reporting of our outcomes. We relied on the trialists defining their compared interventions as shorter and longer-term (or similar terminology). We did not include cluster randomized trials, quasi randomized trials, or observational studies.

### Data extraction and risk of bias assessment

Two review authors (SJ, CKJ) independently screened relevant trials, extracted data using a standardised data extraction sheet, and assessed the risk of bias according to the Risk of Bias (ROB) assessment tool provided in Cochrane Handbook of Systematic Reviews of Interventions [14]. Any discrepancies were resolved through discussion or, if required, through discussion with a third author (JCJ, SS). We contacted trial authors by e-mail if relevant data were unclear or missing. For more information on our risk of bias assessments, see our protocol [6].

### Outcomes and subgroup analyses

Our primary outcomes were quality of life, serious adverse events (as defined by the ICH-GCP guidelines) [15], and symptom severity. Our secondary outcomes were suicide or suicide attempts (dichotomous data), self-harm (dichotomous data), and level of functioning. For all outcomes, we used the trial results reported at the time point closest to the end of treatment in the long-term treatment group.

We planned the following subgroup analyses on our primary outcomes:

- High risk of bias trials compared to low risk of bias trials
- Types of mental health disorders
- Types of psychotherapy comparisons
- Trials above and below the mean difference in intervention lengths

### Assessment of statistical and clinical significance

We performed our meta-analyses according to the recommendations stated in the Cochrane Handbook for Systematic Reviews of Interventions [14], Keus et al. [16], and the eight-step procedure suggested by Jakobsen et al. [17] for better validation of meta-analytic results in systematic reviews. Review Manager 5.4 and Stata 16 were used for all meta-analyses [18, 19]. We planned to use risk ratios (RR) for dichotomous outcomes, mean differences (MD) for continuous outcomes assessed with homogeneous measures, and standardised mean difference (SMD) for continuous outcomes with heterogeneous measures. We reported both the random-effects and the fixed-effect meta-analysis results, but primarily emphasized the most conservative result (highest P value) of the two results, and considered the less conservative results a sensitivity analysis [17]. We used the best–worst/worst-best case scenarios to assess the potential impact of missing outcome data [6, 17]. We planned to use Trial Sequential Analysis to control for random errors and to report Trial Sequental Analysis-adjusted CIs if the cumulative Z-curves did not reach the futility area or passed the diversity-adjusted required information size (DARIS) [6, 17, 20–28]. Trial Sequential Analysis estimates the DARIS (that is the number of participants needed in a meta-analysis to detect or reject a certain intervention effect). When analysing continuous outcomes, we pragmatically anticipated an intervention effect equal to the MD of the observed SD/2 [29]. Heterogeneity was assessed by calculating inconsistency (I^2^) for traditional meta-analyses and diversity (D^2^) for Trial Sequential Analysis. If it was not possible to perform Trial Sequential Analysis to estimate if there was enough information, we calculated the required information size for each single trial result and assessed if there was adequate power to confirm or reject realistic intervention effects of single trial results. For dichotomous outcomes, we used the proportion of participants with an event in the control group, a relative risk reduction of 20%, an alpha of 1.4%, and a beta of 20% as predefined in our protocol [6]. For continuous outcomes, we used the observed mean and standard deviation for the control group, the observed mean in the control group plus or minus the observed standard deviation in the control group/2 for the experimental group, an alpha of 1.4%, and a beta of 20% as predefined in our protocol [6]. We assessed a total of six primary and secondary outcome and, hence, considered a *p*-value of 0.014 as the threshold for statistical significance [17, 30]. We performed independent samples t-tests to calculate *p*-values for single trial results for continuous outcomes, and Fisher’s exact test for single trial results for dichotomous outcomes. We used The Grading of Recommendations Assessment, Development and Evaluation (GRADE) to assess the certainty of evidence [17, 31–33].

## Results

### Study characteristics

**Fig. 1:**
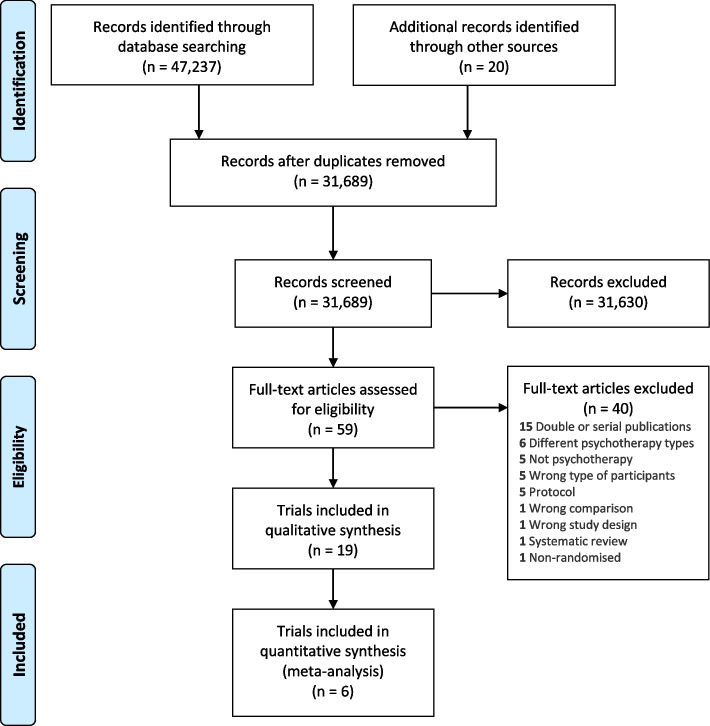
PRISMA flow diagram

On June 27, 2022 our literature search identified a total of 31,689 records after duplicates were removed (Fig. 1). We included 19 randomised clinical trials enrolling a total of 3,447 participants [34–52] (McMain S: The effectiveness of 6 versus 12-months of dialectical behaviour therapy for borderline personality disorder: the feasibility of a shorter treatment and evaluating responses (FASTER) trial, Unpublished) (Supplementary material 3). A list of excludes studies with reasons can be found in Supplementary material 4.

Characteristics of included trials can be found in Table 1. All trials were assessed as at high risk of bias (Supplementary material 5). Five trials assessed the difference between shorter- and longer-term cognitive behavioural therapy for anxiety disorders [36–38, 42, 48]. Four trials assessed the difference between shorter- and longer-term cognitive behavioural therapy for major depressive disorder [39–41, 43]. Three trials assessed the difference between shorter- and longer-term psychodynamic psychotherapy for major depressive disorder [40, 41, 44]. Two trials assessed the difference between shorter- and longer-term psychodynamic psychotherapy for mood- and anxiety disorders [34, 35]. Three trials assessed the difference between shorter- and longer-term prolonged exposure for post-traumatic stress disorder [46, 47, 49]. One trial assessed the difference between shorter- and longer-term interpersonal therapy for major depressive disorder [39]. One trial assessed the difference between shorter- and longer-term cognitive behavioural therapy for post-traumatic stress disorder [45]. One trial assessed the difference between shorter- and longer-term internet-based cognitive behavioural therapy for post-traumatic stress disorder [51]. One factorial trial compared internet-based cognitive behavioral therapy for 8 versus 12 weeks with or without booster sessions for depression and anxiety [50]. One trial assessed the difference between shorter- and longer-term dialectical behavioural therapy for borderline personality disorder [52, 53] (McMain S: The effectiveness of 6 versus 12-months of dialectical behaviour therapy for borderline personality disorder: the feasibility of a shorter treatment and evaluating responses (FASTER) trial, Unpublished).

**Table 1:** Characteristics of included trials

Trial	Country	Number of randomised participants	Mental health disorder	Shorter-term intervention	Longer-term intervention	Overall risk of bias	Primary outcome
**Barkham et al. 1996**^a^ [40]	United Kingdom	54	Major depressive disorder	8 sessions CBT (8 weeks)	16 sessions CBT (18 weeks)	High	No primary outcome was reported
				8 sessions psychodynamic-interpersonal therapy (8 weeks)	16 sessions psychodynamic-interpersonal therapy (18 weeks)		
**Bohni et al. 2009**^a^ [42]	Denmark	48	Panic disorder	8 sessions CBT (3 weeks)	13 sessions CBT (13 weeks)	High	No primary outcome was reported
**Böttche et al. 2021** [51]	Egypt, Saudi Arabia, Algeria, Syria, Morocco, Palestine	224	PTSD	6 sessions internet-based CBT (3 weeks)	10 sessions internet-based CBT (5 weeks)	High	Primary outcome was PTSD symptoms as measured with the Posttraumatic Stress Diagnostic Scale (PDS)
**Bruijniks et al. 2020** [39]	The Netherlands	200	Major depressive disorder	20 sessions CBT (24 weeks)	20 sessions CBT (16 weeks)	High	Primary outcome was depression severity as measured with the BDI-II
				20 sessions interpersonal therapy (24 weeks)	20 sessions interpersonal therapy (16 weeks)		
**Christensen et al. 2006**^a^ [43]	Australia	931	Major depressive disorder	Brief online CBT and problem solving (unclear duration)	Extended online CBT and problem solving (unclear duration)	High	Primary outcome was depression severity as measured with the Goldberg Depression Scale
**Clark et al. 1999** [37]	United Kingdom	29	Panic disorder	5 sessions CBT (12 weeks)	12 sessions CBT (12 weeks)	High	No primary outcome was reported
**Dekker et al. 2005** [44]	The Netherlands	103	Major depressive disorder	8 sessions short psychodynamic supportive psychotherapy (8 weeks)	16 sessions short psychodynamic supportive psychotherapy (24 weeks)	High	No primary outcome was reported
**Dell et al. 2022** [49]	Australia	138	PTSD	10 sessions prolonged exposure (2 weeks)	10 sessions prolonged exposure (10 weeks)	High	Primary outcome was severity of PTSD symptoms assessed with the Clinician- Administered PTSD Scale (CAPS).3
**Ehlers et al. 2014** [45]	United Kingdom	61	PTSD	14 sessions cognitive therapy (5 weeks)	12 sessions cognitive therapy (12 weeks)	High	Primary outcome was severity of PTSD symptoms assessed with the Clinician- Administered PTSD Scale (CAPS)
**Foa et al. 2018** [46]	USA	219	PTSD	10 sessions prolonged exposure therapy (2 weeks)	10 sessions prolonged exposure therapy (8 weeks)	High	Primary outcome was severity of PTSD symptoms assessed with the PTSD Symptom Scale–Interview (PSS-I)
**Hadjistavropoulos et al. 2022** [50]	Canada	434	Depression and/or anxiety	8 sessions internet-based CBT (8 weeks)	11 sessions internet-based CBT (8 weeks + 3 booster sessions)	High	Primary outcomes were severity of depression and anxiety assessed with the Patient Health Questionnaire (PHQ-9) and the Generalized Anxiety Disorder-7 (GAD-7)
				12 sessions internet-based CBT (12 weeks)	15 sessions internet-based CBT (12 weeks + 3 booster sessions)		
**Herbert et al. 2004** [36]	USA	34	Social anxiety disorder	12 sessions CBT (12 weeks)	12 sessions CBT (18 weeks)	High	No primary outcome was reported
**Kenardy et al. 2003** [48]	Australia and Scotland	81^b^	Panic disorder	6 sessions CBT (6 weeks)	12 sessions CBT (12 weeks)	High	Primary measures included a comprehensive battery of panic and anxiety measures
**Knekt et al. 2008** [34]	Finland	229	Mood- and anxiety disorders	20 sessions psychodynamic therapy (20 weeks)	468 sessions psychodynamic therapy (156 weeks)	High	Primary outcomes were depressive and anxiety symptoms
**Lorentzen et al. 2013** [35]	Norway	167	Mood- anxiety- and personality disorders	20 sessions psychodynamic group therapy (20 weeks)	80 sessions psychodynamic group therapy (80 weeks)	High	No primary outcome was reported
**McMain et al**	Canada	240	BPD	26 sessions DBT (26 weeks)	52 sessions DBT (52 weeks)	High	Primary outcome was frequency of suicidal or non-suicidal self-injurious episodes
**Nacasch et al. 2015** [47]	Israel	40	PTSD	10–15 sessions prolonged exposure therapy (60 min)	10–15 sessions prolonged exposure therapy (90 min)	High	Primary outcome was severity of PTSD symptoms assessed with the Posttraumatic Symptom Scale-Interview (PSS-I)
**Roberge et al. 2008** [38]	Canada	65	Panic disorder with agoraphobia	7 sessions CBT (16 weeks)	14 sessions CBT (15 weeks)	High	No primary outcome was reported
**Shapiro et al. 1994**^a^ [41]	United Kingdom	150	Major depressive disorder	8 sessions CBT (8 weeks)	16 sessions CBT (18 weeks)	High	No primary outcome was reported
				8 sessions psychodynamic-relationship-oriented therapy (8 weeks)	16 sessions of psychodynamic-relationship-oriented therapy (18 weeks)		

^a^The results of these trials were not reported in a usable way; i.e. the results were reported in a graph, and standard deviations were not provided for the point estimates

^b^This trial randomised a total of 186 participants to four groups. The number of randomised participants for the two relevant groups were not sufficiently reported, as only the number of participants who commenced treatment was reported

*BPD* Borderline personality disorder, *CBT* Cognitive behavior therapy, *DBT* Dialectical behavior therapy, *PTSD* Post-traumatic stress disorder

All trials compared different durations (weeks of treatment), dosages (number of sessions), and session lengths (minutes) (Table 1). Furthermore, trialists’ definitions of short-term and long-term psychotherapy were not consistent across studies. Most trials compared different numbers of sessions delivered over different durations (e.g. 8 sessions delivered over 8 weeks compared with 16 sessions delivered over 16 weeks) [34, 35, 40–42, 44, 45, 48, 50, 51] (McMain S: The effectiveness of 6 versus 12-months of dialectical behaviour therapy for borderline personality disorder: the feasibility of a shorter treatment and evaluating responses (FASTER) trial, Unpublished). Some trials compared different numbers of sessions delivered over the same duration (e.g. six sessions delivered over 12 weeks compared with 12 sessions delivered over 12 weeks) [37, 38]. Some trials compared the same number of sessions over different durations (e.g. 10 sessions delivered over two weeks compared with 10 sessions delivered over 10 weeks) [36, 39, 46, 49]. Two trials compared the same number of sessions, but with different sessions lengths in minutes (e.g. 10–15 sessions of 60 min compared with 10–15 sessions of 90 min) [43, 47]. We planned to assess serious adverse events. However, only one of the trials reported on this outcome (McMain S: The effectiveness of 6 versus 12-months of dialectical behaviour therapy for borderline personality disorder: the feasibility of a shorter treatment and evaluating responses (FASTER) trial, Unpublished). For several of our review outcomes it was not possible to conduct meta-analysis due to insufficient data. Four trials did not report the results in a usable way [40–43], i.e. they reported the results on a graph and/or did not include standard deviations for each point estimate on a group level. We contacted trial authors to receive relevant data, but we have not received any responses. It was not possible to perform Trial Sequential Analyses to assess the risk of random errors on any of our review outcomes because of lack of relevant data. Only a few trials reported on our dichotomous outcomes, and the continuous outcomes were assessed with heterogeneous measures. We therefore performed sample size calculations for all single trial results to estimate the required information size needed to confirm or reject realistic intervention effects for all outcomes. Results of these sample size calculations can be found in Supplementary material 6.

Due to the large heterogeneity in participants, interventions, and lengths of trials included in this review, we will present the single trial results first. Second, we will present the meta-analysis results.

### Single trial results

#### Trials including participants with borderline personality disorder

We identified one trial randomising 240 participants with borderline personality disorder to six months versus 12 months dialectical behavioral therapy [52, 53] (McMain S: The effectiveness of 6 versus 12-months of dialectical behaviour therapy for borderline personality disorder: the feasibility of a shorter treatment and evaluating responses (FASTER) trial, Unpublished). We retrieved the data through the published trial report and personal communication with the trialists. This trial reported data on all our pre-defined review outcomes. It was not possible to include the trial in a pre-defined meta-analysis, as it was the only trial including participants with borderline personality disorder. The trial reached their pre-calculated sample size of 240 participants [52, 53]. The trial showed no evidence of a difference between short-term and long-term dialectical behavioral therapy when assessing quality of life (*p* = 0.831, required information size reached), serious adverse events (*p* = 1, required information size not reached), symptom severity (*p* = 0.833, required information size reached), suicide or suicide attempts (*p* = 1, required information size not reached), self-harm (*p* = 0.28, required information size not reached), and level of functioning (*p* = 0.731, required information size reached) (Table 2, Supplementary material 6). This trial was assessed as at overall high risk of bias due to lack of blinding of participants and personnel, and due to incomplete outcome data (Supplementary material 5), and the certainty of evidence was assessed as “very low” for all outcomes (Supplementary material 7).

**Table 2:** Single trial results

**Trials including participants with major depressive disorder**^**a**^									
**Trial characteristics**			**Primary review outcomes**			**Secondary review outcomes**			**Trialists’ own conclusions**
**Trial ID**	**Shorter-term intervention**	**Longer-term intervention**	**Quality of Life**	**Serious Adverse Events**	**Symptom severity**	**Suicide/Suicide attempts**	**Self-harm**	**Level of functioning**	
**Barkham et al. 1996** [40]	8 sessions CBT (8 weeks)	16 sessions CBT (18 weeks)	-	-	-	-	-	-	Clients given 16 sessions showed a statistically significant advantage over clients given 8 sessions on some measures at some assessments
	8 sessions psychodynamic-interpersonal therapy (8 weeks)	16 sessions psychodynamic-interpersonal therapy (18 weeks)							
**Bruijniks et al. 2020** [39]	20 sessions CBT (24 weeks)	20 sessions CBT (16 weeks)	CBT: The mean RAND-36 scores at EoT were 50.13 (22.20) for the short-term group (*n* = 49) and 51.53 (22.36) for the long-term group (*n* = 39) (*p* = 0.77) IPT: The mean RAND-36 scores at EoT were 46.8 (20.46) in the short-term group (*n* = 36) and 53.46 (20.67) in the long-term group (*n* = 47) (*p* = 0.14)	-	CBT: The mean (SD) BDI scores at EoT were 24.16 (15.09) for the short-term group (*n* = 37) and 21.25 (12.90) for the long-term group (*n* = 35) (*p* = 0.38) IPT: The mean (SD) BDI scores at EoT were 22.91 (14.75) for the short-term group (*n* = 34) and 20.02 (16.05) for the long-term group (*n* = 39) (*p* = 0.42)	-	-	-	In clinical practice settings, delivery of twice weekly sessions of CBT and IPT for depression was superior to once weekly sessions when assessing depression outcomes
	20 sessions interpersonal therapy (24 weeks)	20 sessions interpersonal therapy (16 weeks)							
**Christensen 2006** [43]	Brief online CBT and problem solving (unclear duration)	Extended online CBT and problem solving (unclear duration)	-	-	-	-	-	-	Brief CBT-based interventions are not as effective as extended interventions
**Dekker et al. 2005** [44]	8 sessions short psychodynamic supportive psychotherapy (8 weeks)	16 sessions short psychodynamic supportive psychotherapy (24 weeks)	The mean (SD) QLDS scores at EoT were 22.6 (8.6) for the short-term group (*n* = 45) and 22.8 (8.3) for the long-term group (*n* = 45) (*p* = 0.911)	-	The mean (SD) HDRS scores at EoT were 11.1 (6.8) for the short-term group (*n* = 45) and 12.1 (7.6) for the long-term group (*n* = 45) (*p* = 0.512)	-	-	-	Eight or 16 psychotherapy sessions in addition to 8 sessions of pharmacotherapy over a period of 6 months would appear to be equally effective in terms of dealing with symptoms
**Shapiro et al. 1994** [41]	8 sessions CBT (8 weeks)	16 sessions CBT (18 weeks)	-	-	-	-	-	-	There is no added benefit from 16 treatment sessions compared with 8
	8 sessions psychodynamic-relationship-oriented therapy (8 weeks)	16 sessions of psychodynamic-relationship-oriented therapy (18 weeks)							
**Trials including participants with anxiety disorders**^**a**^									
**Trial characteristics**			**Primary review outcomes**			**Secondary review outcomes**			**Trialists’ own conclusions**
**Trial ID**	**Shorter-term intervention**	**Longer-term intervention**	**Quality of Life**	**Serious Adverse Events**	**Symptom severity**	**Suicide/Suicide attempts**	**Self-harm**	**Level of functioning**	
**Bohni et al. 2009** [42]	8 sessions CBT (3 weeks)	13 sessions CBT (13 weeks)	-	-	-	-	-	-	Patients in massed CBT achieved their results at a faster rate than patients in spaced CBT, with outcomes after 3 weeks in massed CBT comparable with those achieved after approximately 3 months in spaced CBT
**Clark et al. 1999** [37]	5 sessions CBT (12 weeks)	12 sessions CBT (12 weeks)	-	-	The mean (SD) BAI scores at EoT were 9.8 (6.7) for the short-term group (*n* = 14) and 8.4 (8.0) for the long-term group (*n* = 15) (*p* = 0.615). This result is included in a meta-analysis	-	-	-	Brief CT did not differ from full CT at posttreatment or at follow-up, and effect sizes were essentially the same
**Herbert et al. 2004** [36]	12 sessions CBT (12 weeks)	12 sessions CBT (18 weeks)	-	-	The mean (SD) SPAI-SP scores at EoT were 76.71 (47.18) for the short-term group (n = 15) and 113.77 (39.56) for the long-term group (n = 19) (*p* = 0.018). This result is included in a meta-analysis	-	-	-	The results revealed that the standard treatment program in which therapy was provided over 12 successive weeks resulted in more rapid symptom reduction and lower dropout relative to the extended treatment delivered over 18 weeks
**Kenardy et al. 2003** [48]	6 sessions CBT (6 weeks)	12 sessions CBT (12 weeks)	-	-	The mean (SD) STAI-T scores at EoT were 47.86 (12.31) for the short-term group (*n* = 39) and 41.10 (13.14) for the long-term group (*n* = 42) (*p* = 0.0195). This result is included in a meta-analysis	-	-	-	A brief version performs significantly worse than the standard duration treatment at posttreatment
**Roberge et al. 2008** [38]	7 sessions CBT (16 weeks)	14 sessions CBT (15 weeks)	-	-	The mean (SD) PAS scores at EoT were 10.2 (8.8) for the short-term group (*n* = 32) and 9.5 (10.3) for the long-term group (*n* = 33) (*p* = 0.77). This result is included in a meta-analysis	-	-	-	Brief CBT effectiveness appears comparable to standard CBT in the short term
**Trials including participants with mood- and anxiety disorders**^**a**^									
**Trial characteristics**			**Primary review outcomes**			**Secondary review outcomes**			**Trialists’ own conclusions**
**Trial ID**	**Shorter-term intervention**	**Longer-term intervention**	**Quality of Life**	**Serious Adverse Events**	**Symptom severity**	**Suicide/Suicide attempts**	**Self-harm**	**Level of functioning**	
**Knekt et al. 2008** [34]	Short-term psychodynamic therapy	Long-term psychodynamic therapy	-	-	The mean (SD) HDRS scores at EoT were 10.8 (5.65) for the short-term group (*n* = 83) and 9.0 (6.0) for the long-term group (*n* = 107) (*p* = 0.037)	-	-	The mean (SD) SAS-work scores at EoT were 1.88 (0.55) for the short-term group (*n* = 83) and 1.72 (0.62) for the long-term group (*n* = 107). (*p* = 0.066). This result is included in a meta-analysis	Patients receiving short-term psychodynamic psychotherapy recovered faster from both depressive and anxiety symptoms during the first year of follow-up. During the following 2 years, the symptoms persisted at the level reached in the brief therapy group, whereas in the long-term psychodynamic psychotherapy group the improvement continued during the entire 3-year period. In the long run, long-term psychodynamic psychotherapy thus gave greater benefits than those achieved by the brief therapies
**Lorentzen et al. 2013** [35]	Short-term psychodynamic group therapy	Long-term psychodynamic group therapy	-	-	-	There were 0/77 suicides or suicide attempts in the short-term group compared to 0/90 in the long-term group (*p* = not applicable)	-	The mean (SD) GAF scores at EoT were 67.8 (11.7) for the short-term group (*n* = 71) and 68.1 (14.2) for the long-term group (*n* = 79) (*p* = 0.889). This result is included in a meta-analysis	We observed that short- and long-term therapy were equally effective across 3 years, using IIP, GAF-S and GAF-F as the outcome variables. However, there was a trend in favour of long-term therapy (*P* = 0.10) using GAF-S as the outcome variable
**Hadjistavropoulos et al. 2022** [50]	Internet-based CBT (8 weeks)	Internet-based CBT (8 weeks + 3 booster sessions)	The mean (SD) EQ-5D-5L scores at EoT were 71.92 (18.93) for the short-term group (*n* = 79) and 71.36 (21.34) for the long-term group (*n* = 87) (*p* = 0.858)	-	The mean (SD) PHQ-9 scores at EoT were 7.93 (5.36) for the short-term group (*n* = 79) and 5.84 (5.07) for the long-term group (*n* = 87) (*p* = 0.01) The mean (SD) GAD-7 scores at EoT were 7.56 (5.37) for the short-term group (*n* = 79) and 5.56 (4.60) for the long-term group (*n* = 87) (*p* = 0–01)	-	-	The mean (SD) SDS scores at EoT were 13.86 (7.86) for the short-term group (*n* = 79) and 10.66 (8.7) for the long-term group (*n* = 87) (*p* = 0.01)	No significant group differences were found in this study
	Internet-based CBT (12 weeks)	Internet-based CBT (12 weeks + 3 booster sessions)	The mean (SD) EQ-5D-5L scores at EoT were 74.06 (15.94) for the short-term group (*n* = 87) and 69.13 (21.93) for the long-term group (*n* = 91) (*p* = 0.089)	-	The mean (SD) PHQ-9 scores at EoT were 6.52 (5.23) for the short-term group (*n* = 87) and 7.55 (6.24) for the long-term group (*n* = 91) (*p* = 0.235) The mean (SD) GAD-7 scores at EoT were 6.33 (5.19) for the short-term group (*n* = 87) and 6.96 (5.8) for the long-term group (*n* = 91) (*p* = 0.446)	-	-	The mean (SD) SDS scores at EoT were 10.29 (8.01) for the short-term group (*n* = 87) and 11.39 (8.46) for the long-term group (*n* = 91) (*p* = 0.374)	
**Trials including participants with post-traumatic stress disorder**^**a**^									
**Trial characteristics**			Primary review outcomes			Secondary review outcomes			Trialists’ own conclusions
**Trial ID**	Shorter-term intervention	Longer-term intervention	Quality of Life	Serious Adverse Events	Symptom severity	Suicide/Suicide attempts	Self-harm	Level of functioning	
**Böttche et al. 2021** [51]	6 sessions internet-based CBT (3 weeks)	10 sessions internet-based CBT (5 weeks)	The mean (SD) EUROHIS-QOL-8 scores at EoT were 5.53 (0.83) for the short-term group (*n* = unclear) and 5.11 (1.02) for the long-term group (*n* = unclear) (*p* = *0.75*)	-	The mean (SD) PDS change scores at EoT were -14.73 (1.45) for the short-term group (n = unclear) and -15.03 (1.64) for the long-term group (*n* = unclear) (*p* = 0.89)	-	-	-	The shorter condition results in the same symptom change and dropout rate as the longer condition
**Dell et al. 2022** [49]	Massed prolonged expoure	Standard prolonged exposure	-	0 events	The mean (SD) CAPS scores at EoT were 27.69 (18.42) for the short-term group (*n* = 63) and 25.68 (16.59) for the long-term group (*n* = 71) (*p* = 0.664)	0 events	-	-	Massed prolonged exposure was non-inferior to standard prolonged exposure in reducing symptoms of PTSD
**Ehlers et al. 2014** [45]	Intensive cognitive therapy	Standard cognitive therapy	The mean (SD) Q-LES-Q scores at EoT were 52.67 (20.21) for the short-term group (*n* = 30) and 62.93 (21.70) for the long-term group (*n* = 31) (*p* = 0.061)	-	The mean (SD) CAPS scores at EoT were 32.22 (27.20) for the short-term group (*n* = 30) and 26.97 (28.68) for the long-term group (*n* = 31) (*p* = 0.466)	-	-	The mean (SD) SDS scores at EoT were 9.30 (8.20) for the short-term group (*n* = 30) and 10.02 (9.76) for the long-term group (*n* = 31) (*p* = 0.757)	A novel 7-day intensive version of cognitive therapy for PTSD was well tolerated, achieved faster symptom reduction, and led to comparable overall outcomes as the standard once-weekly cognitive therapy delivered over 3 months
**Foa et al. 2018** [46]	Massed prolonged exposure	Extended prolonged exposure	-	-	The mean (SD) PSS-I scores at EoT were 18.88 (no SD reported) for the short-term group (*n* = 110) and 18.34 (no SD reported) for the long-term group (*n* = 110) (*p* = not applicable)	-	-	-	Among active duty military personnel with PTSD, massed prolonged exposure therapy (10 sessions delivered over 2 weeks) was noninferior to spaced pro- longed exposure therapy (10 sessions delivered over 8 weeks)
**Nacasch et al. 2015** [47]	60 min sessions of prolonged exposure	90 min sessions of prolonged exposure	-	-	The mean (SD) PSS-I scores at EoT were 13.3 (9.52) for the short-term group (*n* = 20) and 12.24 (8.02) for the long-term group (*n* = 17) (*p* = 0.719)	-	-	-	In sum, 20-min imaginal exposure within 60-min sessions yielded noninferior outcomes in PTSD symptoms and posttraumatic negative cognitions at posttreatment and follow-up to the 40-min imaginal exposures and 90-min sessions
**Trials including participants with borderline personality disorder**^**a**^									
**Trial characteristics**			Primary review outcomes			Secondary review outcomes			Trialists’ own conclusions
**Trial**	Shorter-term intervention	Longer-term intervention	Quality of Life	Serious Adverse Events	Symptom severity	Suicide/Suicide attempts	Self-harm	Level of functioning	
**McMain et al. 2022** [52] (McMain S: The effectiveness of 6 versus 12-months of dialectical behaviour therapy for borderline personality disorder: the feasibility of a shorter treatment and evaluating responses (FASTER) trial, Unpublished)	6 months of DBT	12 months of DBT	The mean (SD) overall EQ5DL scores at EoT were 60.7 (21.43) for the short-term group (*n* = 91) compared with 61.41 (23.17) in the long-term group (*n* = 90) (*p* = 0.831)	2 / 90 participants had one or more serious adverse events in the short-term group at EoT compared with 2 / 93 in the long-term group (*p* = 1) (based on suicide/ suicide attempt data only)	The mean (SD) BSL scores at EoT were 38.6 (22.4) for the short-term group (*n* = 90) compared with 39.3 (22.2) in the long-term group (*n* = 91) (*p* = 0.833)	2 / 90 participants had one suicide or suicide-attempts in the short-term group at EoT compared with 2 / 93 in the long-term group (*p* = 1)	28 / 90 participants had one or more deliberate self-harm incidents in the short-term group at EoT compared with 37/ 93 in the long-term group (*p* = 0.28)	The mean (SD) SAS scores at EoT were 2.51 (0.58) for the short-term group (*n* = 90) compared with 2.54 (0.59) in the long-term group (*n* = 91) (*p* = 0.731)	Half the dose of the standard DBT yielded noninferior improvements across time points for the primary outcome, total self-harm frequency, as well as several clinical outcomes

^a^Data is presented for the primary time-point of assessment (end of treatment)

*BAI* Beck Anxiety Inventory, *BSL* Borderline Symptom List-23, *DBT* Dialectical Behavioural Therapy, *CAPS* Clinician Administered PTSD Scale, *CBT* Cognitive Behavioural Therapy, *EoT* End of treatment, *EQ5DL* Euroqol-5D-5, *HDRS* Hamilton Depression Rating Scale, *IPT* Interpersonal Therapy, *PAS* Panic and Agoraphobia Scale, *PSS-I* PTSD Symptom Scale Interview, *SAS* Social Adjustment Scale, *SD* Standard deviation, *SDS* Sheehan Disability Scale, *SPAI-SP* Social Phobia Anxiety Inventory – Social Phobia, *STAI-T* State Trait Anxiety Inventory-Trait, *QLDS* Quality of Life Depression Scale, *Q-LES-Q* Quality of Life Enjoyment and Satisfaction Questionnaire

#### Trials including participants with mood- and anxiety disorders

We identified three trials assessing the effects of shorter- versus longer-term psychotherapy for mood- and anxiety disorders [34, 35, 50].

One trial randomising 229 participants with mood- and anxiety disorders to 20 weeks versus 156 weeks of psychodynamic psychotherapy [34] showed no evidence of a difference when assessing symptom severity (*p* = 0.037, required information size reached), considering our adjusted threshold for significance was pre-defined at 0.014 in our protocol [6], or level of functioning (*p* = 0.066, required information size reached). The trial almost reached their sample size (230 participants) [34], but it was unclear whether this sample size was pre-defined. One trial randomising 167 participants with mood- and anxiety disorders to 20 weeks versus 80 weeks of psychodynamic psychotherapy [35] showed no evidence of a difference when assessing the proportion of participants with a suicide or a suicide attempts (zero events in both groups) or level of functioning (*p* = 0.889, required information size not reached) (Table 2, Supplementary material 6). Both trials were assessed at high risk of bias (Supplementary material 5) and the certainty of evidence was assessed as “very low” for all outcomes (Supplementary material 8). These two trials are included in a meta-analysis (see below).

We also identified one factorial trial randomising 496 participants with major depressive disorder and anxiety disorders to internet-based cognitive behavioral therapy for 8 versus 12 weeks with or without 3 booster sessions [50]. This trial showed no evidence of a difference when assessing quality of life for either of the two pairwise comparisons (8 weeks versus 8 weeks plus boosters *p* = 0.858; 12 weeks versus 12 weeks plus boosters* p* = 0.089; required information size reached). The trial showed evidence of a beneficial effect of adding booster sessions in both pairwise comparisons when assessing symptom severity (8 weeks versus 8 weeks plus boosters *p* = 0.01; 12 weeks versus 12 weeks plus boosters* p* = 0.01; required information size reached) and level of functioning (8 weeks versus 8 weeks plus boosters *p* = 0.01; 12 weeks versus 12 weeks plus boosters* p* = 0.01; required information size reached) (Table 2, Supplementary material 6). Both trials were assessed at high risk of bias (Supplementary material 5), and the certainty of evidence was assessed as “very low” for all outcomes (Supplementary materials 9 and 10).

#### Trials including participants with major depressive disorder

We identified five trials including eight comparisons assessing the effects of shorter- versus longer-term psychotherapy for participants with major depressive disorder [39–41, 43, 44]. Four trials compared shorter- versus longer-term cognitive behavioural therapy for major depressive disorder [39–41, 43]. Three trials compared shorter- versus longer-term psychodynamic psychotherapy for major depressive disorder [40, 41, 44]. One trial compared shorter- versus longer-term interpersonal therapy for major depressive disorder [39]. It was not possible to perform meta-analyses, as the trials differed in the assessed psychotherapy traditions, and only two trials reported on our pre-defined review outcomes [39, 44].

One trial randomising 200 participants with major depressive disorder to once- versus twice weekly cognitive behavioral therapy or interpersonal therapy [39] showed no evidence of a difference when assessing quality of life and symptom severity for either cognitive behavioral therapy (*p* = 0.77 and *p* = 0.38, required information size not reached) or interpersonal therapy (*p* = 0.14 and *p* = 0.42, required information size not reached). One trial randomising 103 participants with major depressive disorder to eight versus 16 sessions of short-term psychodynamic supportive psychotherapy [44] showed no evidence of a difference when assessing quality of life (*p* = 0.911, required information size not reached) or symptom severity (*p* = 0.512, required information size not reached) (Table 2, Supplementary material 6). Both trials were assessed at high risk of bias (Supplementary material 5) and the certainty of evidence was assessed as “very low” for all outcomes (Supplementary materials 11, 12, and 13).

### Trials including participants with post-traumatic stress disorder

We identified five trials assessing the effects of shorter- versus longer-term psychotherapy for participants with post-traumatic stress disorder [45–47, 49, 51]. Three trials compared shorter- versus longer-term prolonged exposure for post-traumatic stress disorder [46, 47, 49]. One trial compared shorter- versus longer-term cognitive behavioral therapy for post-traumatic stress disorder [45]. One trial compared shorter- versus longer-term internet-based cognitive behavioral therapy for post-traumatic stress disorder [51]. It was not possible to perform meta-analyses, as the trials differed in the assessed psychotherapy traditions, and one of them did not report standard deviations [46]. The two remaining trials reported on some of our pre-defined review outcomes.

One trial randomising 224 participants with post-traumatic stress disorder to 6 versus 10 assignments of internet-based cognitive behavioral therapy showed no evidence of a difference when assessing quality of life (*p* = 0.75, required information size not reached) and symptom severity (*p* = 0.89, required information size not reached) [51]. One trial randomising 138 participants with post-traumatic stress disorder to massed prolonged exposure (10 sessions delivered over 2 weeks) versus standard prolonged exposure (10 sessions delivered over 10 weeks) showed no evidence of a difference when assessing symptom severity (*p* = 0.664; required information size not reached) [49]. One similar trial did not report standard deviations, but the trialists concluded that massed prolonged exposure therapy (10 sessions delivered over 2 weeks) was noninferior to spaced prolonged exposure therapy (10 sessions delivered over 8 weeks) [46]. One trial randomising 61 participants with post-traumatic stress disorder to intensive (5 weeks) versus standard (12 weeks) cognitive therapy [45] showed no evidence of a difference when assessing quality of life (*p* = 0.061, required information size not reached), symptom severity (*p* = 0.466, required information size not reached), or level of functioning (*p* = 0.757, required information size not reached). One trial randomising 40 participants with post-traumatic stress disorder to 60 min versus 90 min sessions of prolonged exposure therapy [47] showed no evidence of a difference when assessing symptom severity (*p* = 0.719, required information size not reached) (Table 2, Supplementary material 6). All trials were assessed at high risk of bias (Supplementary material 5) and the certainty of evidence was assessed as “very low” for all outcomes (Supplementary materials 14, 15, and 16).

#### Trials including participants with anxiety disorders

We identified five trials assessing the effects of shorter- versus longer-term cognitive behavioral therapy for anxiety disorders [36–38, 42, 48]. One trial did not report the results in a usable way; i.e. the results were reported on a graph and standard deviations were not reported [42].

One trial randomising 29 participants with panic disorder to five versus 12 sessions cognitive behavioral therapy [37] showed no evidence of a difference when assessing symptom severity (*p* = 0.615, required information size not reached). One trial randomising 34 participants with social anxiety disorder to 12 versus 18 weeks of cognitive behavioral therapy [36] showed no evidence of a difference when assessing symptom severity (*p* = 0.018, required information size not reached), considering our adjusted threshold for significance was pre-defined at 0.014 in our protocol [6]. One trial randomising 81 participants to six versus 12 weeks of cognitive behavioral therapy for participants with panic disorder [48] showed no evidence of a difference when assessing symptom severity (*p* = 0.0195, required information size not reached), considering our adjusted threshold for significance was pre-defined at 0.014 in our protocol [6]. One trial randomising 65 participants with panic disorder and agoraphobia to 7 sessions versus 14 sessions cognitive behavioral therapy [38] showed no evidence of a difference when assessing symptom severity (*p* = 0.77, required information size not reached). All trials were assessed at high risk of bias (Supplementary material 5) and the certainty of evidence was assessed as “very low” for all outcomes (Supplementary material 17).

It was only possible to perform two pre-planned meta-analyses: one assessing the effects of shorter- versus longer-term cognitive behavioral therapy for anxiety disorders at end of treatment and at maximum follow-up, and another one assessing the effects of shorter- versus longer-term psychodynamic psychotherapy for mood and anxiety disorders at end of treatment.

### Shorter- versus longer-term cognitive behavioural therapy for anxiety disorders

We identified five trials assessing the effects of shorter- versus longer-term cognitive behavioural therapy for anxiety disorders [36–38, 42, 48]. All trials were assessed as at high risk of bias (Supplementary material 5). One trial was not eligible for meta-analysis, as the results were not reported in a usable way; i.e. the results were reported on a graph and standard deviations were not reported [42].

Four trials randomising a total of 209 participants reported on anxiety symptoms [36–38, 48]. Four different symptom scales were used: Beck Anxiety Inventory (BAI) [37], Social Phobia Anxiety Inventory – Social Phobia [36], State Trait Anxiety Inventory-Trait (STAI-T) [48], and Panic and Agoraphobia Scale (PAS) [38]. One trial included participants with social anxiety disorder [36]. Two trials included participants with panic disorder [37, 48]. One trial included participants with panic disorder and agoraphobia [38]. We chose to analyse anxiety symptoms using SMD.

#### Meta-analysis of anxiety symptoms at end of treatment

**Fig. 2:**
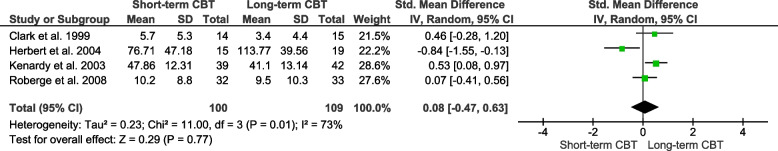
Forest plot of shorter- versus longer-term cognitive behavioural therapy for anxiety disorders on severity of anxiety symptoms at end of treatment

Random-effects meta-analysis showed no evidence of a difference between shorter (5, 6, 7, 12 weeks) and longer-term (12, 12, 14, 18 weeks) cognitive behavioural therapy for anxiety disorders (including social anxiety disorder, panic disorder, and panic disorder with agoraphobia) on anxiety symptoms at end of treatment (SMD: 0.08; 95% CI: -0.47 to 0.63; *p* = 0.77; I^2^ = 73%; four trials; very low certainty) (Fig. 2). Visual inspection of the forest plot and measures to quantify heterogeneity indicated substantial heterogeneity (I^2^ = 73%). The end of treatment assessment time point was 12 weeks [37, 48], 15 weeks [38], and 18 weeks [36]. It was not possible to assess the possible impact of missing outcome data, due to unclear or lack of reporting of number of analysed participants in some of the included trials. It was not possible to perform Trial Sequential Analysis for this outcome, because the outcome was assessed using SMD [24]. This outcome result was assessed as at high risk of bias. Certainty of the evidence was assessed as ‘very low’. See Supplementary material 17. The fixed-effect meta-analysis showed similar results (SMD 0.16; 95% CI: -0.11, 0.44; *p* = 0.25; I^2^ = 73%; four trials; very low certainty) Supplementary material 18.

### Shorter- versus longer-term psychodynamic therapy for mood and anxiety disorders

We identified two trials assessing the effects of shorter- versus longer-term psychodynamic therapy for mood- and anxiety disorder [34, 35, 54]. Both trials were assessed as at high risk of bias (Supplementary material 4).

Two trials randomising a total of 393 participants reported on level of functioning [34, 35]. Two different assessment scales were used, including Global Assessment of Functioning – Function (GAF-F) [35] and the work subscale (SAS-Work) of the Social Adjustment Scale [34]. We chose to analyze level of functioning using standardised mean difference. In order to assure the scales pointed in the right direction, we multiplied the mean in one of the trials with ‘-1’.

#### Meta-analysis of level of functioning at end of treatment

**Fig. 3:**
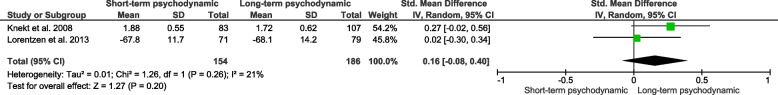
Forest plot of shorter- versus longer-term psychodynamic therapy for mood- and anxiety disorders on level of functioning at end of treatment

Random effects meta-analysis showed no evidence of a difference between shorter- (20 and 20 weeks) and longer-term (80 and 156 weeks) psychodynamic psychotherapy for mood and anxiety disorders on level of functioning at end of treatment (SMD 0.16; 95% CI -0.08 to 0.40; *p* = 0.20; I^2^ = 21%; two trials; very low certainty) (Fig. 3). Visual inspection of forest plot and measures to quantify heterogeneity (I^2^ = 21%) showed some heterogeneity. The end of treatment time point of assessment was 36 months after randomisation for both trials. It was not possible to perform Trial Sequential Analysis for this outcome, because the outcome was assessed using SMD [24]. This outcome result was assessed as at high risk of bias. Certainty of the evidence was assessed as ‘very low’. See Supplementary material 8. The fixed-effect meta-analysis showed similar results (SMD 0.16; 95% CI: -0.05, 0.37; *p* = 0.14; I^2^ = 21%; two trials; very low certainty) Supplementary material 19.

#### Incomplete outcome data

Random effects meta-analysis of the best–worst case scenario adding 2 SD (SMD -0.16; 95% CI -8.13 to 7.81; *p* =  < 0.00001; I^2^ = 95%) and adding 1 SD (SMD -0.15; 95% CI -4.26 to 3.95; *p* =  < 0.94; I^2^ = 100%) for missing data showed no evidence of a difference between shorter- and longer-term psychodynamic psychotherapy. Random effects meta-analysis of the worst-best case scenario adding 2 SD (SMD -0.14; 95% CI -7.62 to 7.35; *p* =  < 0.97; I^2^ = 100%) and adding 1 SD (SMD -0.14; 95% CI -3.76 to 3.48; *p* =  < 0.94; I^2^ = 100%) for missing values showed no evidence of a difference between shorter- and longer-term psychodynamic psychotherapy.

Because of lack of relevant data, it was not possible to conduct other pre-defined meta-analyses. It was only possible to perform one sensitivity analysis (best–worst worst-best scenarios) to assess the potential impact of incomplete outcome data. We also planned several subgroup analyses to test for heterogeneity [6], but it was not possible to conduct them because of lack of relevant data. Further, it was not possible to assess the risk of publication bias by testing for funnel plot asymmetry due to lack of trials. Last, it was not possible to perform Trial Sequential Analyses because all included outcomes were assessed using SMD.

### The possible contribution of ongoing trials

We identified two ongoing trials [55, 56] that might contribute to the current evidence on shorter- versus longer-term psychotherapy for adult mental health disorders. These ongoing trials will contribute to the evidence on quality of life, serious adverse events, symptom severity, suicide and suicide attempts, self-harm, and level of functioning.

## Discussion

We conducted the first systematic review assessing the difference between shorter- and longer-term psychotherapy for adult mental health disorders. We included 19 trials randomising a total of 3,447 participants to a shorter or a longer-term version of the same psychotherapy type. All trials and outcome results were at high risk of bias, and the certainty of the evidence according to GRADE was `very low' for all outcomes.

One single trial showed no evidence of a difference between shorter- versus longer-term dialectical behavioral therapy for borderline personality disorder and reached the required information size needed to confirm or reject realistic intervention effects when assessing quality of life, symptom severity, and level of functioning [53] (McMain S: The effectiveness of 6 versus 12-months of dialectical behaviour therapy for borderline personality disorder: the feasibility of a shorter treatment and evaluating responses (FASTER) trial, Unpublished). One single trial showed evidence of a beneficial effect of adding booster sessions to 8 and 12 weeks of internet-based cognitive-behavioral therapy when assessing symptom severity and level of functioning and reached the required information size needed to confirm or reject realistic intervention effects [50]. One single trial showed no evidence of a difference between shorter- versus longer-term psychodynamic psychotherapy for mood- or anxiety disorders and reached the required information size needed to confirm or reject realistic intervention effects when assessing symptom severity and level of functioning [34]. The remaining single trials did not meet the required information size needed to confirm or reject realistic intervention effects. It was only possible to perform two pre-planned meta-analyses. Meta-analysis showed no evidence of a difference between short-term and long-term cognitive behavioural therapy for anxiety symptoms at end of treatment or at maximum follow-up. Meta-analysis showed no evidence of a difference between short-term and long-term psychodynamic psychotherapy on level of functioning at end of treatment. All trials and outcomes were assessed as at high risk of bias, and the certainty of evidence was assessed as ‘very low’ for all outcomes. It was not possible to perform Trial Sequential Analysis or tests for publication bias. Further, due to poor reporting in the included trials, we only performed one planned sensitivity analysis to assess the potential impact of missing data. Only one trial reported on serious adverse events (McMain S: The effectiveness of 6 versus 12-months of dialectical behaviour therapy for borderline personality disorder: the feasibility of a shorter treatment and evaluating responses (FASTER) trial, Unpublished). Two trials reported on suicide and suicide attempts [35] (McMain S: The effectiveness of 6 versus 12-months of dialectical behaviour therapy for borderline personality disorder: the feasibility of a shorter treatment and evaluating responses (FASTER) trial, Unpublished), and one trial reported on self-harm (McMain S: The effectiveness of 6 versus 12-months of dialectical behaviour therapy for borderline personality disorder: the feasibility of a shorter treatment and evaluating responses (FASTER) trial, Unpublished).

Our review has several strengths. We followed our protocol which was registered prior to the systematic literature search (PROSPERO ID: CRD42019128535). Data were double-extracted by independent authors minimizing the risk of inaccurate data extraction, and we assessed the risk of bias in all trials according to Cochrane methodology [14]. We used GRADE to assess the certainty of the evidence [31–33], and the eight-step assessment suggested by Jakobsen et al. to assess if the thresholds for significance were crossed [17]. Hence, this systematic review considered both risks of random errors and risks of systematic errors which adds further robustness to our results and conclusions. Another strength of our review is that we pragmatically accepted any short-term psychotherapy type and any long-term psychotherapy type, thus results may therefore guide a clinician when choosing between different treatment durations.

Our review also has several limitations. First, due to large heterogeneity in participants, interventions, comparisons, and outcomes, we decided to primarily report the results narratively and only perform two small pre-planned meta-analyses. The observed heterogeneity is due to our pre-defined broad inclusion criteria, i.e. we used the trialists’ own definitions of short-term and long-term psychotherapy. However, we believe that this choice of methodology from a pragmatic point of view is the best solution there is, as introducing specific thresholds may have excluded important data from our review [6]. If we had used a specific threshold distinguishing short-term from long-term psychotherapy, e.g. by applying a definition of short-term psychotherapy as including up to 24 sessions and long-term psychotherapy as including at least 50 sessions or having a duration of at least one year as suggested by others [57, 58], we would have only been able to include three trials in the review, and the aim of presenting a complete overview would not be possible. Second, all trials were at high risk of bias. Therefore, there is a risk that our results overestimated the beneficial effects and underestimated the harmful effects of the experimental interventions being studied [59–66]. Third, we only identified 19 trials, and it was not possible to assess the risk of random errors in the meta-analyses with Trial Sequential Analysis due to the inclusion of continuous outcomes assessed with heterogeneous measures (i.e. we assessed the effects with standardised mean difference). This is a major limitation, as we cannot assess if the shown lack of difference is an indication of a “true” lack of difference, or if it is an indication that more trials are needed. We calculated the required information sizes for single trial results post-hoc, but these should primarily be considered exploratory, as they rely on the observed means and standard deviations instead of pre-defined minimal clinically important differences on the assessed scales. Fourth, only few trials reported on serious adverse events, suicide, suicide attempts, and self-harm. It is of utmost importance to always assess beneficial *and* harmful intervention effects on patient-important outcomes [14, 67].

We have identified one previous systematic review comparing short-term and long-term psychotherapy for schizophrenia [68]. However, the review did not identify any trials. We have also identified a meta-regression study investigating the effects of psychotherapy for major depressive disorder [5]. This study found no significant association between the duration of psychotherapy and effect-size, which is similar to the conclusion of the present review. However, in the meta-regression study, there was a strong association between number of sessions per week and effect size. An increase from one to two sessions per week increased the effect size with *g* = 0.45, while keeping the total number of treatment sessions constant [5]. The results of the present review could neither confirm nor reject that two sessions per week were more efficacious than one session per week.

The included trials in this review typically assessed the effects of different durations of psychotherapy for anxiety disorders, major depressive disorder, and post-traumatic stress disorder. Our findings indicate that there may be no evidence of a difference between short-term and long-term psychotherapy when assessing symptom severity and level of functioning. There are, however, indications from non-controlled studies that patients with complex and severe psychopathology, defined by the presence of, e.g., co-occurring mental health disorders, longer duration and early onset of the disorder, and unemployment, may have better outcomes in high-intensity than in low-intensity treatments [69, 70]. We included one trial including participants with borderline personality disorder. This trial did not find evidence of a difference between six versus 12 months dialectical behavioral therapy, and the trial reached the required information size needed to confirm or reject realistic intervention effects for quality of life, symptom severity, and level of functioning. However, the trial was assessed as at high risk of bias and the certainty of evidence was “very low” for all outcomes. Accordingly, future randomised clinical trials comparing the outcomes of short- and long-term psychotherapy for patients with low and high problem complexity should be conducted. We are currently performing a similar randomised clinical trial assessing the effects of five months versus 14 months of mentalization-based therapy for borderline personality disorder [55, 71]. We are planning a protocol for an individual patient data meta-analysis of shorter- versus longer-term psychotherapy for borderline personality disorder, which will be conducted once data from the two trials become available. Results of the individual patient data meta-analysis will increase the possibility of identifying subgroups of participants with specific effects of the assessed interventions. We identified no trials including participants with other severe personality pathology, schizophrenia, or other psychotic disorders. Hence, it is still unclear whether patients with severe psychopathology requires short-term or long-term psychotherapy.

Evidence-based practice and decision-making should be based on the best available evidence, patient preferences, and the clinician’s expertise [72]. For severe and complex cases there is evidence of beneficial effects of psychotherapy of specific treatment lengths (e.g. long-term specialized treatment for borderline personality disorder [73]) but very low certainty evidence to guide clinicians in choosing the optimal treatment duration. Evidently, clinicians should by default offer psychotherapy in a duration supported by the best available evidence. But when there is a question of treatment duration, e.g. a patient asking for a shorter treatment because of life circumstances, the clinician is advised to balance this preference with clinical experience which may include knowledge of specific prognostic factors such as early onset or co-occurring disorders, while also considering the poor evidence regarding the optimal treatment duration currently available.

## Conclusions

The evidence for shorter- versus longer-term psychotherapy for adult mental health disorders is currently unclear. We only identified 19 randomised clinical trials. More trials at low risk of bias and at low risk of random errors assessing participants at different levels of psychopathological severity are urgently needed.

### Differences between the protocol and the review

In addition to assessing all outcomes at end of treatment, we planned to assess all outcomes at maximum follow-up as a secondary analysis. However, only few trials reported data at maximum follow-up. Because of lack of relevant data, we chose to only report data at end of treatment.

## Supplementary Information

**Additional file 1.** PRISMA 2020 Checklist for “Short-term versus long-term psychotherapy for adult psychiatric disorders: A systematic review with meta-analysis”

**Additional file 2.** 

**Additional file 3.** 

**Additional file 4.** 

**Additional file 5:Supplementary material 5.** Risk of bias table.

**Additional file 6.** 

**Additional file 7.** 

**Additional file 8.** 

**Additional file 9.** 

**Additional file 10.** 

**Additional file 11.** 

**Additional file 12.** 

**Additional file 13.** 

**Additional file 14.** 

**Additional file 15.** 

**Additional file 16.** 

**Additional file 17.** 

**Additional file 18:Supplementary material 18.** Fixed-effect meta-analysis of short-term versus long-term cognitivebehavioral therapy for anxiety disorders on anxiety symptoms (sensitivity analysis).

**Additional file 19:Supplementary material 19.** Fixed-effect meta-analysis of short-term versus long-term psychodynamic psychotherapy for mood- and anxiety disorders on level of functioning (sensitivity analysis).

## Data Availability

All data generated or analysed during this study are included in this published article (and its supplementary information files).

## Abbreviations

CENTRAL: Cochrane Central Register of Controlled Trials
CI: Confidence Interval
CPCI-S: Conference Proceedings Citation Index—Science
CPCI-SSH: Conference Proceedings Citation Index—Social Science & Humanities
DARIS: Diversity Adjusted Required Information Size
EMBASE: Excerpta Medica database
GRADE: Grades of Recommendation, Assessment, Development, and Evaluation
LILACS: Latin American and Caribbean Health Sciences Literature
MEDLINE: Medical Literature Analysis and Retrieval System Online
MD: Mean Difference
PRISMA: Preferred Reporting Items for Systematic Reviews and Meta-Analysis
ROB: Risk of Bias
RR: Risk Ratio
SCI-EXPANDED: Science Citation Index Expanded
SD: Standard Deviation
SMD: Standardised Mean Difference
SSCI: Social Sciences Citation Index
TSA: Trial Sequential Analysis
