# Supplementary material for: The difference between shorter- versus longer-term psychotherapy for adult mental health disorders: a systematic review with meta-analysis
Source: BMC Psychiatry. 2023 Jun 16;23:438. doi: 10.1186/s12888-023-04895-6 (PMC10273498; doi:10.1186/s12888-023-04895-6)
Supplement: Supplementary file 2 — Additional file 2. [file 12888_2023_4895_MOESM2_ESM.doc]

**Search strategies for**

**Short-term versus long-term psychotherapy for adult psychiatric disorders: a protocol for a systematic review with meta-analysis and Trial Sequential Analysis**

**(S Juul)**

**Search performed 27 June 2022**

**Total number of records identified: 47237 records**

**Number of duplicates excluded: 15568 records**

**Number of records in final list: 31669 records**

**Cochrane Central Register of Controlled Trials (CENTRAL; 2022, Issue 6) in the Cochrane Library (17247 hit)**

#1 MeSH descriptor: [Attention Deficit Disorder with Hyperactivity] explode all trees

#2 ("attention deficit hyperactivity disorder" or adhd):ti,ab

#3 #1 or #2

#4 MeSH descriptor: [Psychotic Disorders] explode all trees

#5 MeSH descriptor: [Schizophrenia] explode all trees

#6 (psychotic or delusion* or hallucination* or ((disorgani*ed or abnormal) NEAR (think* or motor*)) or schizophreni*):ti,ab

#7 #4 or #5 or #6

#8 MeSH descriptor: [Bipolar Disorder] explode all trees

#9 (bipolar or "mood elevation" or mania or hypomania or depress*):ti,ab

#10 #8 or #9

#11 MeSH descriptor: [Depressive Disorder] explode all trees

#12 (depressi* or mood or unipolar):ti,ab

#13 #11 or #12

#14 MeSH descriptor: [Anxiety Disorders] explode all trees

#15 (anxiet* or fear or "avoidance behavior*" or phobia* or panic* or agoraphobia):ti,ab

#16 #14 or #15

#17 MeSH descriptor: [Obsessive-Compulsive Disorder] explode all trees

#18 ("obsessive compulsive disorder" or OCD or urge* or obsessi* or (repetetive NEAR (mental or behavior*))):ti,ab

#19 #17 or #18

#20 MeSH descriptor: [Stress Disorders, Post-Traumatic] explode all trees

#21 (post-trauma* or trauma*):ti,ab

#22 #20 or #21

#23 MeSH descriptor: [Feeding and Eating Disorders] explode all trees

#24 ("eating behavior" or anorexia* or bulimia* or binge-eating*):ti,ab

#25 #23 or #24

#26 MeSH descriptor: [Personality Disorders] explode all trees

#27 (schizotypal* or paranoid* or schizoid* or histrionic* or narcissistic* or antisocial* or borderline* or avoidant* or dependent or obsessive-compulsi*):ti,ab

#28 #26 or #27

#29 3 or 7 or 10 or 13 or 16 or 19 or 22 or 25 or 28

#30 MeSH descriptor: [Psychotherapy] explode all trees

#31 (((psycho* or cognitive or behavior* or humanistic or systemic) NEAR therap*) or psychotherap* or "self care" or self-care):ti,ab

#32 #30 or #31

#33 (brief or extended or standard or intensiv* or ((short* or long*) NEXT term)):ti,ab

#34 #32 and #33

#35 #29 and #34

**MEDLINE Ovid (1946 to 27 June 2022) (6964 hits)**1. exp Attention Deficit Disorder with Hyperactivity/

2. (attention deficit hyperactivity disorder or adhd).ti,ab.

3. 1 or 2

4. exp Psychotic Disorders/

5. exp Schizophrenia/

6. (psychotic or delusion* or hallucination* or ((disorgani*ed or abnormal) adj (think* or motor*)) or schizophreni*).ti,ab.

7. 4 or 5 or 6

8. exp Bipolar Disorder/

9. (bipolar or mood elevation or mania or hypomania or depress*).ti,ab.

10. 8 or 9

11. exp Depressive Disorder/

12. (depressi* or mood or unipolar).ti,ab.

13. 11 or 12

14. exp Anxiety Disorders/

15. (anxiet* or fear or avoidance behavior* or phobia* or panic* or agoraphobia).ti,ab.

16. 14 or 15

17. exp Obsessive-Compulsive Disorder/

18. (obsessive compulsive disorder or OCD or urge* or obsessi* or (repetetive adj (mental or behavior*))).ti,ab.

19. 17 or 18

20. exp Stress Disorders, Post-Traumatic/

21. (post-trauma* or trauma*).ti,ab.

22. 20 or 21

23. exp "Feeding and Eating Disorders"/

24. (eating behavior or anorexia* or bulimia* or binge-eating*).ti,ab.

25. 23 or 24

26. exp Personality Disorders/

27. (schizotypal* or paranoid* or schizoid* or histrionic* or narcissistic* or antisocial* or borderline* or avoidant* or dependent or obsessive-compulsi*).ti,ab.

28. 26 or 27

29. 3 or 7 or 10 or 13 or 16 or 19 or 22 or 25 or 28

30. exp psychotherapy/

31. (((psycho* or cognitive or behavior* or humanistic or systemic) and therap*) or psychotherap* or self care or self-care).ti,ab.

32. 30 or 31

33. (brief or extended or standard or intensiv* or ((short* or long*) and term)).mp. [mp=title, abstract, original title, name of substance word, subject heading word, floating sub-heading word, keyword heading word, organism supplementary concept word, protocol supplementary concept word, rare disease supplementary concept word, unique identifier, synonyms]

34. 32 and 33

35. 29 and 34

36. limit 35 to ("all adult (19 plus years)" or "adolescent (13 to 18 years)" or "young adult (19 to 24 years)" or "adult (19 to 44 years)" or "young adult and adult (19-24 and 19-44)" or "middle age (45 to 64 years)" or "middle aged (45 plus years)" or "all aged (65 and over)" or "aged (80 and over)")

37. (random* or blind* or placebo* or meta-analys*).mp. [mp=title, abstract, original title, name of substance word, subject heading word, floating sub-heading word, keyword heading word, organism supplementary concept word, protocol supplementary concept word, rare disease supplementary concept word, unique identifier, synonyms]

38. 36 and 37

**Embase Ovid (1974 to 27 June 2022) (8773 hits)**

1. exp attention deficit disorder/

2. (attention deficit hyperactivity disorder or adhd).ti,ab.

3. 1 or 2

4. exp psychosis/

5. exp schizophrenia/

6. (psychotic or delusion* or hallucination* or ((disorgani*ed or abnormal) adj (think* or motor*)) or schizophreni*).ti,ab.

7. 4 or 5 or 6

8. exp bipolar disorder/

9. (bipolar or mood elevation or mania or hypomania or depress*).ti,ab.

10. 8 or 9

11. exp depression/

12. (depressi* or mood or unipolar).ti,ab.

13. 11 or 12

14. exp anxiety disorder/

15. (anxiet* or fear or avoidance behavior* or phobia* or panic* or agoraphobia).ti,ab.

16. 14 or 15

17. exp obsessive compulsive disorder/

18. (obsessive compulsive disorder or OCD or urge* or obsessi* or (repetetive adj (mental or behavior*))).ti,ab.

19. 17 or 18

20. exp posttraumatic stress disorder/

21. (post-trauma* or trauma*).ti,ab.

22. 20 or 21

23. exp eating disorder/

24. (eating behavior or anorexia* or bulimia* or binge-eating*).ti,ab.

25. 23 or 24

26. exp personality disorder/

27. (schizotypal* or paranoid* or schizoid* or histrionic* or narcissistic* or antisocial* or borderline* or avoidant* or dependent or obsessive-compulsi*).ti,ab.

28. 26 or 27

29. 3 or 7 or 10 or 13 or 16 or 19 or 22 or 25 or 28

30. exp psychotherapy/

31. (((psycho* or cognitive or behavior* or humanistic or systemic) and therap*) or psychotherap* or self care or self-care).ti,ab.

32. 30 or 31

33. (brief or extended or standard or intensiv* or ((short* or long*) and term)).mp. [mp=title, abstract, original title, name of substance word, subject heading word, floating sub-heading word, keyword heading word, organism supplementary concept word, protocol supplementary concept word, rare disease supplementary concept word, unique identifier, synonyms]

34. 32 and 33

35. 29 and 34

36. limit 35 to (adult <18 to 64 years> or aged <65+ years>)

37. (random* or blind* or placebo* or meta-analys*).mp. [mp=title, abstract, heading word, drug trade name, original title, device manufacturer, drug manufacturer, device trade name, keyword, floating subheading word, candidate term word]

38. 36 and 37

**LILACS (Bireme; 1982 to 27 June 2022) (1578 hits)**

(attention deficit hyperactivity disorder or adhd) or (psychotic or delusion$ or hallucination$ or ((disorgani$ed or abnormal) and (think$ or motor$)) or schizophreni$) or (bipolar or mood elevation or mania or hypomania or depress$) or (depressi$ or mood or unipolar) or (anxiet$ or fear or avoidance behavior$ or phobia$ or panic$ or agoraphobia) or (obsessive compulsive disorder or OCD or urge$ or obsessi$ or (repetetive and (mental or behavior$))) or (post-trauma$ or trauma$) or (eating behavior or anorexia$ or bulimia$ or binge-eating$) or (schizotypal$ or paranoid$ or schizoid$ or histrionic$ or narcissistic$ or antisocial$ or borderline$ or avoidant$ or dependent or obsessive-compulsi$) [Words] and (((psycho$ or cognitive or behavior$ or humanistic or systemic) and therap$) or psychotherap$ or self care or self-care) [Words] and (brief or extended or standard or intensiv$ or ((short$ or long$) and term)) [Words]

**PsycINFO (EBSCO*host*; 1806 to 27 June 2022) (5877 hits)**

S37 S35 AND S36

S36 TX (random* or blind* or placebo* or meta-analys*)

S35 S29 AND S34

S34 S32 AND S33

S33 TI ( (brief or extended or standard or intensiv* or ((short* or long*) and term)) ) OR AB ( (brief or extended or standard or intensiv* or ((short* or long*) and term)) )

S32 S30 OR S31

S31 TI ( (((psycho* or cognitive or behavior* or humanistic or systemic) and therap*) or psychotherap* or self care or self-care) ) OR AB ( (((psycho* or cognitive or behavior* or humanistic or systemic) and therap*) or psychotherap* or self care or self-care) )

S30 MA Psychotherapy

S29 S3 OR S7 OR S10 OR S13 OR S16 OR S19 OR S22 OR S25 OR S28

S28 S26 OR S27

S27 TI ( (schizotypal* or paranoid* or schizoid* or histrionic* or narcissistic* or antisocial* or borderline* or avoidant* or dependent or obsessive-compulsi*) ) OR AB ( (schizotypal* or paranoid* or schizoid* or histrionic* or narcissistic* or antisocial* or borderline* or avoidant* or dependent or obsessive-compulsi*) )

S26 MA Personality Disorders

S25 S23 OR S24

S24 TI ( (eating behavior or anorexia* or bulimia* or binge-eating*) ) OR AB ( (eating behavior or anorexia* or bulimia* or binge-eating*) )

S23 MA Feeding and Eating Disorders

S22 S20 OR S21

S21 TI ( (post-trauma* or trauma*) ) OR AB ( (post-trauma* or trauma*) )

S20 MA Stress Disorders, Post-Traumatic

S19 S17 OR S18

S18 TI ( (obsessive compulsive disorder or OCD or urge* or obsessi* or (repetetive near (mental or behavior*))) ) OR AB ( (obsessive compulsive disorder or OCD or urge* or obsessi* or (repetetive near (mental or behavior*))) )

S17 MA Obsessive-Compulsive Disorder

S16 S14 OR S15

S15 TI ( (anxiet* or fear or avoidance behavior* or phobia* or panic* or agoraphobia) ) OR AB ( (anxiet* or fear or avoidance behavior* or phobia* or panic* or agoraphobia) )

S14 MA Anxiety Disorders

S13 S11 OR S12

S12 TI ( (depressi* or mood or unipolar) ) OR AB ( (depressi* or mood or unipolar) )

S11 MA Depressive Disorder

S10 S8 OR S9

S9 TI ( (bipolar or mood elevation or mania or hypomania or depress*) ) OR AB ( (bipolar or mood elevation or mania or hypomania or depress*) )

S8 MA Bipolar Disorder

S7 S4 OR S5 OR S6

S6 TI ( (psychotic or delusion* or hallucination* or ((disorgani*ed or abnormal) near (think* or motor*)) or schizophreni*) ) OR AB ( (psychotic or delusion* or hallucination* or ((disorgani*ed or abnormal) near (think* or motor*)) or schizophreni*) )

S5 MA Schizophrenia

S4 MA Psychotic Disorders

S3 S1 OR S2

S2 TI ( (attention deficit hyperactivity disorder or adhd) ) OR AB ( (attention deficit hyperactivity disorder or adhd) )

S1 MA attention deficit disorder with hyperactivity

**Science Citation Index Expanded (SCI-EXPANDED) (1900 to 27 June 2022); Social Sciences Citation Index (SSCI) (1956 to 27 June 2022); Conference Proceedings Citation Index- Science (CPCI-S) (1990 to 27 June 2022); and Conference Proceedings Citation Index- Social Science & Humanities (CPCI-SSH) (1990 to 27 June 2022) (Web of Science) (6798 hits)**

#7 #6 AND #5

#6 TS=(random* or blind* or placebo* or meta-analys*)

#5 #4 AND #1

#4 #3 AND #2

#3 TS=(brief or extended or standard or intensiv* or ((short* or long*) and term))

#2 TS=(((psycho* or cognitive or behavior* or humanistic or systemic) and therap*) or psychotherap* or self care or self-care)

#1 TI=((attention deficit hyperactivity disorder or adhd) or (psychotic or delusion* or hallucination* or ((disorgani*ed or abnormal) and (think* or motor*)) or schizophreni*) or (bipolar or mood elevation or mania or hypomania or depress*) or (depressi* or mood or unipolar) or (anxiet* or fear or avoidance behavior* or phobia* or panic* or agoraphobia) or (obsessive compulsive disorder or OCD or urge* or obsessi* or (repetetive near (mental or behavior*))) or (post-trauma* or trauma*) or (eating behavior or anorexia* or bulimia* or binge-eating*) or (schizotypal* or paranoid* or schizoid* or histrionic* or narcissistic* or antisocial* or borderline* or avoidant* or dependent or obsessive-compulsi*))
