# Supplementary material for: The difference between shorter- versus longer-term psychotherapy for adult mental health disorders: a systematic review with meta-analysis
Source: BMC Psychiatry. 2023 Jun 16;23:438. doi: 10.1186/s12888-023-04895-6 (PMC10273498; doi:10.1186/s12888-023-04895-6)
Supplement: Supplementary file 3 — Additional file 3. [file 12888_2023_4895_MOESM3_ESM.docx]

**List of included papers**

(1, 2, 3, 4, 5, 6, 7, 8, 9, 10, 11, 12, 13, 14, 15, 16, 17, 18, 19)

1. Knekt P, Lindfors O, Harkanen T, Valikoski M, Virtala E, Laaksonen MA, et al. Randomized trial on the effectiveness of long-and short-term psychodynamic psychotherapy and solution-focused therapy on psychiatric symptoms during a 3-year follow-up. Psychol Med. 2008;38(5):689-703.

2. Lorentzen S, Ruud T, Fjeldstad A, Hoglend P. Comparison of short- and long-term dynamic group psychotherapy: randomised clinical trial. Br J Psychiatry. 2013;203(3):280-7.

3. Herbert JD, Rheingold AA, Gaudiano BA, Myers VH. Standard Versus Extended Cognitive Behavior Therapy for Social Anxiety Disorder: A Randomized-Controlled Trial. Behavioural and Cognitive Psychotherapy. 2004;32(2):131-47.

4. Clark DM, Salkovskis PM, Hackmann A, A. W, Ludgate J, Gelder M. Brief Cognitive Therapy for Panic Disorder: A Randomized Controlled Trial. Journal of Consulting and Clinical Psychology. 1999;67(4):583-9.

5. Roberge P, Marchand A, Reinharz D, Savard P. Cognitive-Behavioral Treatment for Panic Disorder With Agoraphobia A Randomized, Controlled Trial and Cost-Effectiveness Analysis. Journal of Behavior Modification. 2008;32(3):333-51.

6. Bruijniks SJE, Lemmens L, Hollon SD, Peeters F, Cuijpers P, Arntz A, et al. The effects of once- versus twice-weekly sessions on psychotherapy outcomes in depressed patients. Br J Psychiatry. 2020;216(4):222-30.

7. Barkham M, Rees A, Shapiro DA, Stiles WB, Agnew RM, Halstead J, et al. Outcomes of time-limited psychotherapy in applied settings: replicating the Second Sheffield Psychotherapy Project. J Consult Clin Psychol. 1996;64(5):1079.

8. Shapiro DA, Barkham M, Rees A, Hardy GE, Reynolds S, Startup M. Effects of treatment duration and severity of depression on the effectiveness of cognitive-behavioral and psychodynamic-interpersonal psychotherapy. J Consult Clin Psychol. 1994;62(3):522.

9. Bohni M, Spindler H, Arendt M, Hougaard E, Rosenberg N. A randomized study of massed three‐week cognitive behavioural therapy schedule for panic disorder. Acta Psychiatr Scand. 2009;120(3):187-95.

10. Christensen H, Griffiths K, Mackinnon A, Brittliffe K. Online randomized controlled trial of brief and full cognitive behaviour therapy for depression. Psychol Med. 2006;36(12):1737.

11. Dekker J, Molenaar PJ, Kool S, Van Aalst G, Peen J, de Jonghe F. Dose–effect relations in time-limited combined psycho-pharmacological treatment for depression. Psychol Med. 2005;35(1):47-58.

12. Ehlers A, Hackmann A, Grey N, Wild J, Liness S, Albert I, et al. A randomized controlled trial of 7-day intensive and standard weekly cognitive therapy for PTSD and emotion-focused supportive therapy. Am J Psychiatry. 2014;171(3):294-304.

13. Foa EB, McLean CP, Zang Y, Rosenfield D, Yadin E, Yarvis JS, et al. Effect of prolonged exposure therapy delivered over 2 weeks vs 8 weeks vs present-centered therapy on PTSD symptom severity in military personnel: A randomized clinical trial. JAMA. 2018;319(4):354-64.

14. Nacasch N, Huppert JD, Su Y-J, Kivity Y, Dinshtein Y, Yeh R, et al. Are 60-Minute Prolonged Exposure Sessions With 20-Minute Imaginal Exposure to Traumatic Memories Sufficient to Successfully Treat PTSD? A Randomized Noninferiority Clinical Trial. Behav Ther. 2015;46(3):328-41.

15. Kenardy JA, Dow MG, Johnston DW, Newman MG, Thomson A, Taylor CB. A comparison of delivery methods of cognitive-behavioral therapy for panic disorder: an international multicenter trial. J Consult Clin Psychol. 2003;71(6):1068.

16. Böttche M, Wagner B, Vöhringer M, Heinrich M, Stein J, Selmo P, et al. Is only one cognitive technique also effective? Results from a randomized controlled trial of two different versions of an internet-based cognitive behavioural intervention for post-traumatic stress disorder in Arabic-speaking countries. Eur J Psychotraumatol. 2021;12(1):1943870.

17. Hadjistavropoulos H, Peynenburg V, Thiessen D, Nugent M, Karin E, Dear B, et al. A randomized factorial trial of internet-delivered cognitive behavioural therapy: An 8-week program with or without extended support and booster lesson. Internet Interventions. 2022;27:100499.

18. Dell L, Sbisa AM, Forbes A, O'Donnell M, Bryant R, Hodson S, et al. Effect of massed v. standard prolonged exposure therapy on PTSD in military personnel and veterans: a non-inferiority randomised controlled trial. Psychol Med. 2022:1-8.

19. McMain SF, Chapman AL, Kuo JR, Dixon-Gordon KL, Guimond TH, Labrish C, et al. The effectiveness of 6 versus 12 months of dialectical behavior therapy for borderline personality disorder: a noninferiority randomized clinical trial. Psychother Psychosom. 2022:1-16.
