# Supplementary material for: The difference between shorter- versus longer-term psychotherapy for adult mental health disorders: a systematic review with meta-analysis
Source: BMC Psychiatry. 2023 Jun 16;23:438. doi: 10.1186/s12888-023-04895-6 (PMC10273498; doi:10.1186/s12888-023-04895-6)
Supplement: Supplementary file 4 — Additional file 4. [file 12888_2023_4895_MOESM4_ESM.docx]

**Excluded studies**

1. Laaksonen, M. A., Knekt, P., & Lindfors, O. (2013). Psychological predictors of the recovery from mood or anxiety disorder in short-term and long-term psychotherapy during a 3-year follow-up. *Psychiatry Research*, *208*(2), 162-173. **(Double or serial publication)**
2. Knekt, P., Laaksonen, M. A., Raitasalo, R., Haaramo, P., & Lindfors, O. (2010). Changes in lifestyle for psychiatric patients three years after the start of short-and long-term psychodynamic psychotherapy and solution-focused therapy. *European psychiatry*, *25*(1), 1-7. **(Double or serial publication)**
3. Knekt, P., Heinonen, E., Härkäpää, K., Järvikoski, A., Virtala, E., Rissanen, J., ... & Helsinki Psychotherapy Study Group. (2015). Randomized trial on the effectiveness of long-and short-term psychotherapy on psychosocial functioning and quality of life during a 5-year follow-up. *Psychiatry Research*, *229*(1-2), 381-388. **(Double or serial publication)**
4. Baker, A., Turner, A., Kay-Lambkin, F. J., & Lewin, T. J. (2009). The long and the short of treatments for alcohol or cannabis misuse among people with severe mental disorders. *Addictive behaviors*, *34*(10), 852-858. **(Different psychotherapy types)**
5. Albert, N., Melau, M., Jensen, H., Emborg, C., Jepsen, J. R. M., Fagerlund, B., ... & Nordentoft, M. (2017). Five years of specialised early intervention versus two years of specialised early intervention followed by three years of standard treatment for patients with a first episode psychosis: randomised, superiority, parallel group trial in Denmark (OPUS II). *Bmj*, *356*. **(Not psychotherapy)**
6. Tecic, T., Schneider, A., Althaus, A., Schmidt, Y., Bierbaum, C., Lefering, R., ... & Neugebauer, E. A. (2011). Early short-term inpatient psychotherapeutic treatment versus continued outpatient psychotherapy on psychosocial outcome: a randomized controlled trial in trauma patients. *Journal of Trauma and Acute Care Surgery*, *70*(2), 433-441. **(Wrong type of participants)**
7. Lorentzen, S., Fjeldstad, A., Ruud, T., & Høglend, P. A. (2015). Comparing short-and long-term group therapy: Seven-year follow-up of a randomized clinical trial. *Psychotherapy and psychosomatics*, *84*(5), 320-322. **(Double or serial publication)**
8. Heinonen, E., Knekt, P., Härkänen, T., Virtala, E., & Lindfors, O. (2018). Childhood adversities as predictors of improvement in psychiatric symptoms and global functioning in solution-focused and short-and long-term psychodynamic psychotherapy during a 5-year follow-up. *Journal of affective disorders*, *235*, 525-534. **(Double or serial publication)**
9. Knekt, P., Virtala, E., Härkänen, T., Vaarama, M., Lehtonen, J., & Lindfors, O. (2016). The outcome of short-and long-term psychotherapy 10 years after start of treatment. *Psychological medicine*, *46*(6), 1175-1188. **(Double or serial publication)**
10. Dow, M., Kenardy, J., Johnston, D., Newman, M., Taylor, C., & Thomson, A. (2007). Prognostic indices with brief and standard CBT for panic disorder: II. Moderators of outcome. Psychological Medicine, 37(10), 1503-1509. **(Double or serial publication)**
11. Knekt, P., Lindfors, O., Sares-Jäske, L., Virtala, E., & Härkänen, T. (2013). Randomized trial on the effectiveness of long-and short-term psychotherapy on psychiatric symptoms and working ability during a 5-year follow-up. *Nordic journal of psychiatry*, *67*(1), 59-68. **(Double or serial publication)**
12. Laurenssen, E. M., Westra, D., Kikkert, M. J., Noom, M. J., Eeren, H. V., van Broekhuyzen, A. J., ... & Dekker, J. J. (2014). Day Hospital Mentalization-Based Treatment (MBT-DH) versus treatment as usual in the treatment of severe borderline personality disorder: protocol of a randomized controlled trial. *BMC psychiatry*, *14*(1), 1-13. **(Protocol)**
13. Ordman, A. M., & Kirschenbaum, D. S. (1985). Cognitive-behavioral therapy for bulimia: An initial outcome study. Journal of Consulting and Clinical Psychology, 53(3), 305–313. **(Different psychotherapy types)**
14. Kool, M., Van, H. L., Bartak, A., de Maat, S. C., Arntz, A., van den Eshof, J. W., ... & Dekker, J. J. (2018). Optimizing psychotherapy dosage for comorbid depression and personality disorders (PsyDos): a pragmatic randomized factorial trial using schema therapy and short-term psychodynamic psychotherapy. *BMC psychiatry*, *18*, 1-15. **(Protocol)**
15. Ben-Itzhak, S., Bluvstein, I., Schreiber, S., Aharonov-Zaig, I., Maor, M., Lipnik, R., & Bloch, M. (2012). The effectiveness of brief versus intermediate duration psychodynamic psychotherapy in the treatment of adjustment disorder. *Journal of Contemporary Psychotherapy*, *42*, 249-256. **(Wrong type of participants)**
16. Botella, C., & García-Palacios, A. (1999). The possibility of reducing therapist contact and total length of therapy in the treatment of panic disorder. *Behavioural and Cognitive Psychotherapy*, *27*(3), 231-247. **(Different psychotherapy types)**
17. Juul, S., Lunn, S., Poulsen, S., Sørensen, P., Salimi, M., Jakobsen, J. C., ... & Simonsen, S. (2019). Short-term versus long-term mentalization-based therapy for outpatients with subthreshold or diagnosed borderline personality disorder: a protocol for a randomized clinical trial. *Trials*, *20*, 1-10. **(Protocol)**
18. De Jong, K., Timman, R., Hakkaart-Van Roijen, L., Vermeulen, P., Kooiman, K., Passchier, J., & Busschbach, J. V. (2014). The effect of outcome monitoring feedback to clinicians and patients in short and long-term psychotherapy: A randomized controlled trial. *Psychotherapy Research*, *24*(6), 629-639. **(Wrong comparison)**
19. Murray, L. K., Haroz, E. E., Doty, B., Singh, N. S., Bogdanov, S., Bass, J., ... & Bolton, P. (2018). Testing the effectiveness and implementation of a brief version of the Common Elements Treatment Approach (CETA) in Ukraine: a study protocol for a randomized controlled trial. *Trials*, *19*(1), 1-16. **(Protocol)**
20. Gottheil, E., Weinstein, S. P., Sterling, R. C., Lundy, A., & Serota, R. D. (1998). A randomized controlled study of the effectiveness of intensive outpatient treatment for cocaine dependence. *Psychiatric Services*, *49*(6), 782-787. **(Different psychotherapy types)**
21. Fjeldstad, A., Høglend, P., & Lorentzen, S. (2017). Patterns of change in interpersonal problems during and after short-term and long-term psychodynamic group therapy: A randomized clinical trial. *Psychotherapy Research*, *27*(3), 350-361. **(Double or serial publication)**
22. Barkham, M., Rees, A., Stiles, W. B., Shapiro, D. A., Hardy, G. E., & Reynolds, S. (1996). Dose–effect relations in time-limited psychotherapy for depression. Journal of Consulting and Clinical Psychology, 64(5), 927–935. **(Wrong study design)**
23. Glick, I. D., Hargreaves, W. A., Drues, J., & Showstack, J. A. (1976). Short versus long hospitalization: A prospective controlled study: IV. One-year follow-up results for schizophrenic patients. *The American Journal of Psychiatry*. **(Not psychohterapy)**
24. Reneses, B., Figuera, D., Salcedo, G., Trujillo, M., López-Ibor, J. J., Galián, M., ... & Serrano, R. (2011). A controlled randomized study on the efficacy of short-term dinamic psychotherapy in borderline personality disorders (BPD). Preliminary results. *European Psychiatry*, *26*(S2), 1040-1040. (**Different psychotherapy types)**
25. Covi, L., Hess, J. M., Schroeder, J. R., & Preston, K. L. (2002). A dose response study of cognitive behavioral therapy in cocaine abusers. *Journal of substance abuse treatment*, *23*(3), 191-197. **(Wrong type of participants)**
26. Naeem, F., Farooq, S., & Kingdon, D. (2014). Cognitive behavioral therapy (brief vs standard duration) for schizophrenia. *Schizophrenia bulletin*, *40*(5), 958-959. (**Systematic review)**
27. Buonocore, M., Bosia, M., Bechi, M., Spangaro, M., Cavedoni, S., Cocchi, F., ... & Cavallaro, R. (2017). Is longer treatment better? A comparison study of 3 versus 6 months cognitive remediation in schizophrenia. *Neuropsychology*, *31*(4), 467. **(Not psychotherapy)**
28. Marchand, A., Roberge, P., Primiano, S., & Germain, V. (2009). A randomized, controlled clinical trial of standard, group and brief cognitive-behavioral therapy for panic disorder with agoraphobia: A two-year follow-up. *Journal of Anxiety Disorders*, *23*(8), 1139-1147. **(Double or serial publication)**
29. McMain, S. F., Chapman, A. L., Kuo, J. R., Guimond, T., Streiner, D. L., Dixon-Gordon, K. L., ... & Hoch, J. S. (2018). The effectiveness of 6 versus 12-months of dialectical behaviour therapy for borderline personality disorder: the feasibility of a shorter treatment and evaluating responses (FASTER) trial protocol. *BMC psychiatry*, *18*(1), 1-16. **(Protocol)**
30. Kashner, T. M., Henley, S. S., Golden, R. M., Rush, A. J., & Jarrett, R. B. (2007). Assessing the preventive effects of cognitive therapy following relief of depression: a methodological innovation. *Journal of affective disorders*, *104*(1-3), 251-261. **(Different psychotherapy types)**
31. Lorentzen, S., Ruud, T., Fjeldstad, A., & Høglend, P. A. (2015). Personality disorder moderates outcome in short‐and long‐term group analytic psychotherapy: A randomized clinical trial. *British Journal of Clinical Psychology*, *54*(2), 129-146. **(Double or serial** **publication)**
32. Knekt, P., Lindfors, O., Laaksonen, M. A., Raitasalo, R., Haaramo, P., Järvikoski, A., & Helsinki Psychotherapy Study Group. (2008). Effectiveness of short-term and long-term psychotherapy on work ability and functional capacity—a randomized clinical trial on depressive and anxiety disorders. *Journal of affective disorders*, *107*(1-3), 95-106. **(Double or** **serial publication)**
33. Knekt, P., Lindfors, O., Laaksonen, M. A., Renlund, C., Haaramo, P., Härkänen, T., ... & Helsinki Psychotherapy Study Group. (2011). Quasi-experimental study on the effectiveness of psychoanalysis, long-term and short-term psychotherapy on psychiatric symptoms, work ability and functional capacity during a 5-year follow-up. *Journal of Affective Disorders*, *132*(1-2), 37-47. **(Double or serial publication)**
34. Barrowclough, C., Marshall, M., Gregg, L., Fitzsimmons, M., Tomenson, B., Warburton, J., & Lobban, F. (2014). A phase-specific psychological therapy for people with problematic cannabis use following a first episode of psychosis: a randomized controlled trial. *Psychological Medicine*, *44*(13), 2749-2761. **(Wrong type of participants)**
35. Hargreaves, W. A., Glick, I. D., Drues, J., Showstack, J. A., & Feigenbaum, E. (1977). Short vs long hospitalization: A prospective controlled study: VI. Two-year follow-up results for schizophrenics. *Archives of General Psychiatry*, *34*(3), 305-311. **(Not psychotherapy)**
36. Kroska, E. B., Roche, A. I., & O'Hara, M. W. (2020). How much is enough in brief Acceptance and Commitment Therapy? A randomized trial. *Journal of Contextual Behavioral Science*, *15*, 235-244. **(Wrong type of participants)**
37. Johnson, D. R., & Lubin, H. (2002). Effect of brief versus long-term inpatient treatment on homecoming stress in combat-related posttraumatic stress disorder: three-year follow-up. *The Journal of nervous and mental disease*, *190*(1), 47-51. **(Non-randomised)**
38. Joutsenniemi, K., Laaksonen, M. A., Knekt, P., Haaramo, P., & Lindfors, O. (2012). Prediction of the outcome of short-and long-term psychotherapy based on socio-demographic factors. *Journal of affective disorders*, *141*(2-3), 331-342. **(Double or serial publication)**
39. Chang, W. C., Chan, G. H. K., Jim, O. T. T., Lau, E. S. K., Hui, C. L. M., Chan, S. K. W., ... & Chen, E. Y. H. (2015). Optimal duration of an early intervention programme for first-episode psychosis: randomised controlled trial. *The British Journal of Psychiatry*, *206*(6), 492-500. **(Not psychotherapy)**
40. Dow, M. G., Kenardy, J. A., Johnston, D. W., Newman, M. G., Taylor, C. B., & Thomson, A. (2007). Prognostic indices with brief and standard CBT for panic disorder: I. Predictors of outcome. *Psychological Medicine*, *37*(10), 1493-1502. **(Double or serial publication)**
