# Supplementary material for: The difference between shorter- versus longer-term psychotherapy for adult mental health disorders: a systematic review with meta-analysis
Source: BMC Psychiatry. 2023 Jun 16;23:438. doi: 10.1186/s12888-023-04895-6 (PMC10273498; doi:10.1186/s12888-023-04895-6)
Supplement: Supplementary file 5 — Additional file 5:Supplementary material 5. Risk of bias table. [file 12888_2023_4895_MOESM5_ESM.pdf]

Supplementary material 5. Risk of bias table

Risk of bias

Study	D1	D2	D3	D4	D5	D6	D7	Overall
Barkham et al. 1996	-	-	-	-	-	-	+	X
Bohni et al. 2009	-	-	-	X	X	-	+	X
Böttche et al. 2021	+	-	-	-	X	+	+	X
Bruijniks et al. 2020	+	+	-	-	X	X	+	X
Christensen et al. 2006	-	-	-	-	-	-	+	X
Clark et al. 1999	-	-	-	-	-	-	X	X
Dekker et al. 2004	-	-	X	+	X	-	+	X
Dell et al. 2022	+	+	X	+	+	+	+	X
Ehlers et al. 2014	-	-	X	+	+	-	+	X
Foa et al. 2018	-	-	-	+	X	X	+	X
Hadjistavropoulos et al. 2022	+	-	X	X	X	+	+	X
Herbert et al. 2004	-	-	X	X	X	-	+	X
Kenardy et al. 2003	-	-	-	-	-	X	X	X
Knekt et al. 2008	+	+	-	X	X	-	+	X
Lorentzen et al. 2013	X	-	-	-	X	X	+	X
McMain et al. 2022	+	+	X	+	X	+	+	X
Nacasch et al. 2015	+	+	-	+	X	-	+	X
Roberge et al. 2008	-	-	-	-	-	-	+	X
Shapiro et al. 1990	-	-	-	-	X	-	+	X

D1: Random sequence generation
D2: Allocation concealment
D3: Blinding of participants and personnel
D4: Blinding of outcome assessment
D5: Incomplete outcome data
D6: Selective reporting
D7: Other sources of bias

Judgement
X High
- Unclear
+ Low
