# Supplementary material for: The difference between shorter- versus longer-term psychotherapy for adult mental health disorders: a systematic review with meta-analysis
Source: BMC Psychiatry. 2023 Jun 16;23:438. doi: 10.1186/s12888-023-04895-6 (PMC10273498; doi:10.1186/s12888-023-04895-6)
Supplement: Supplementary file 6 — Additional file 6. [file 12888_2023_4895_MOESM6_ESM.docx]

|  | | **Predefined review outcomes** | | | | | | | | | | | | |
| --- | --- | --- | --- | --- | --- | --- | --- | --- | --- | --- | --- | --- | --- | --- |
| **Trial** | **Number randomized** | **Quality of life** | | **Serious adverse events** | | **Symptom severity** | | | **Suicide/ suicide-attempts** | | **Self-harm** | | **Level of functioning** | |
| **Barkham et al. 1996** | 54 | - | | - | | - | | | - | | - | | - | |
| **Böttche et al. 2021** | 224 | RIS: | 176 |  | | RIS | 204 | |  | |  | |  | |
| **Bohni et al. 2009*** | 48 | - | | - | | - | | | - | | - | | - | |
| **Bruijniks et al. 2020** | CBT: 100 | CBT: | | - | | CBT: | | | - | | - | | - | |
|  |  | RIS: | 188 |  |  | RIS: | | 190 |  |  |  |  |  |  |
|  | IPT: 100 | IPT | |  |  | IPT: | | |  |  |  |  |  |  |
|  |  | RIS: | 186 |  |  | RIS: | | 178 |  |  |  |  |  |  |
| **Christensen et al. 2006** | 931 | - | | - | | - | | | - | | - | | - | |
| **Clark et al. 1999** | 29 | - | | - | | RIS: | | 148 | - | | - | | - | |
| **Dekker et al. 2004** | 103 | RIS: | 174 | - | | RIS: | | 154 | - | | - | | - | |
| **Dell et al. 2021** | 135 |  | |  | | RIS: | | 170 |  | |  | |  | |
| **Ehlers et al. 2014** | 61 | RIS: | 174 | - | | RIS: | | 188 | - | | - | | RIS: | 170 |
| **Foa et al. 2018** | 219 | - | | - | | N/A | | | - | | - | | - | |
| **Hadjistavropoulos et al. 2022** | ICBT no booster versus booster: 236 | RIS: | 202 | - | | RIS PTSD:  RIS GAD: | | 178  170 | - | |  | | RIS: | 210 |
|  | ICBT-extention no booster versus booster: 233 | RIS: | 182 |  |  | RIS PTSD:  RIS GAD: | | 174  172 |  |  |  |  | RIS: | 174 |
| **Herbert et al. 2004** | 34 | - | | - | | RIS: | | 178 | - | | - | | - | |
| **Kenardy et al. 2003** | 81 | - | | - | | RIS: | | 188 | - | | - | | - | |
| **Knekt et al. 2008** | 229 | - | | - | | RIS: | | 178 | - | | - | | RIS: | 218 |
| **Lorentzen et al. 2013** | 167 | - | | - | | - | | | N/A | | - | | RIS: | 188 |
| **McMain et al. (unpublished data)** | 240 | RIS: | 170 | RIS: | 49,084 | RIS: | | 172 | RIS: | 49,084 | RIS: | 1,596 | RIS: | 172 |
| **Nacasch et al. 2015** | 40 | - | | - | | RIS: | | 162 | - | | - | | - | |
| **Roberge et al. 2008** | 65 | - | | - | | RIS: | | 232 | - | | - | | - | |
| **Shapiro et al. 1990** | 150 | - | | - | | - | | | - | | - | | - | |

CBT; Cognitive Behavioural Therapy: IPT; Interpersonal Therapy: RIS; Required Information Size
