# Supplementary material for: The difference between shorter- versus longer-term psychotherapy for adult mental health disorders: a systematic review with meta-analysis
Source: BMC Psychiatry. 2023 Jun 16;23:438. doi: 10.1186/s12888-023-04895-6 (PMC10273498; doi:10.1186/s12888-023-04895-6)
Supplement: Supplementary file 14 — Additional file 14. [file 12888_2023_4895_MOESM14_ESM.docx]

Summary of Findings table

| **Short-term versus long-term cognitive behavioral therapy for post-traumatic stress disorder at end of treatment** | | | | | | |
| --- | --- | --- | --- | --- | --- | --- |
| **Patients or population:** Post-traumatic stress disorder  **Setting:** Any setting  **Intervention:** Short-term cognitive behavioral therapy  **Comparison:** Long-term cognitive behavioral therapy | | | | | | |
| **Outcomes** | **Anticipated absolute effects** | | **Relative effect (95% CI)** | **No of participants (studies)** | **Certainty of the evidence (GRADE)** | **Comments** |
|  | **Risk with**  **Long-term CBT** | **Risk with**  **Short-term CBT** |  |  |  |  |
| **Quality of life**  Follow-up: 14 weeks | - | - | MD: -10  (-21.01 to 0.49) | 61  (1 RCT) | ⨁◯◯◯ VERY LOW ^a,b,c^ |  |
| **Serious adverse events** | - | **-** | - | - | - | Outcome not yet measured or reported |
| **Symptom severity**  Follow-up: 14 weeks | - | - | MD: 5.25  (-9.07 to 19.57) | 61  (1 RCT) | ⨁◯◯◯ VERY LOW ^a,b,c^ |  |
| **Suicide or suicide attempts** | - | - | - | - | - | Outcome not yet measured or reported |
| **Self-harm** | - | - | - | - | - | Outcome not yet measured or reported |
| **Level of functioning**  Follow-up: 14 weeks | - | - | MD: -0.72  (-5.34 to 3.90) | 61  (1 RCT) | ⨁◯◯◯ VERY LOW ^a,b,c^ |  |
| **CBT:** cognitive behavioral therapy; **CI:** Confidence interval; **GRADE:** GRADE Working Group grades of evidence, **MD:** Mean Difference | | | | | | |
| **GRADE Working Group grades of evidence**  **High certainty:** We are very confident that the true effect lies close to that of the estimate of the effect **Moderate certainty:** We are moderately confident in the effect estimate: The true effect is likely to be close to the estimate of the effect, but there is a possibility that it is substantially different **Low certainty:** Our confidence in the effect estimate is limited: The true effect may be substantially different from the estimate of the effect **Very low certainty:** We have very little confidence in the effect estimate: The true effect is likely to be substantially different from the estimate of effect | | | | | | |

**Explanations**

a. Downgraded 2 for risk of bias

b. Downgraded 1 for imprecision due to low number of participants

c. Downgraded 1 for indirectness because the results are from a single trial from a single country, therefore results may not be generalizable to other settings
