# Supplementary material for: The difference between shorter- versus longer-term psychotherapy for adult mental health disorders: a systematic review with meta-analysis
Source: BMC Psychiatry. 2023 Jun 16;23:438. doi: 10.1186/s12888-023-04895-6 (PMC10273498; doi:10.1186/s12888-023-04895-6)

Supplementary material 18. Fixed-effect meta-analysis of short-term versus long-term cognitive-behavioral therapy for anxiety disorders on anxiety symptoms (sensitivity analysis)

	Short-term CBT			Long-term CBT				Std. Mean Difference
**Study or Subgroup**	**Mean**	**SD**	**Total**	**Mean**	**SD**	**Total**	**Weight**	**IV, Fixed, 95% CI**
Clark et al. 1999	5.7	5.3	14	3.4	4.4	15	13.9%	0.46 [-0.28, 1.20]
Herbert et al. 2004	76.71	47.18	15	113.77	39.56	19	15.1%	-0.84 [-1.55, -0.13]
Kenardy et al. 2003	47.86	12.31	39	41.1	13.14	42	38.7%	0.53 [0.08, 0.97]
Roberge et al. 2008	10.2	8.8	32	9.5	10.3	33	32.2%	0.07 [-0.41, 0.56]
**Total (95% CI)**			**100**			**109**	**100.0%**	**0.16 [-0.11, 0.44]**

Heterogeneity: Chi² = 11.00, df = 3 (P = 0.01); I² = 73%

Test for overall effect: Z = 1.16 (P = 0.25)

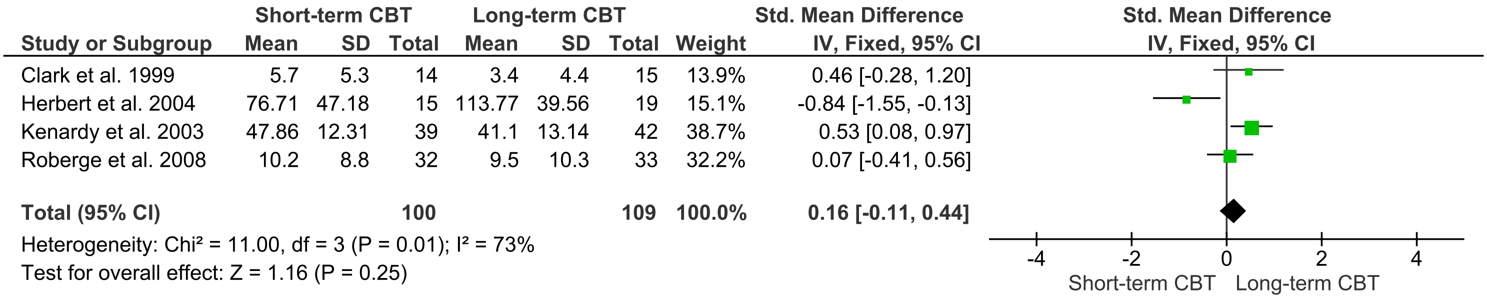

Supplement: Supplementary file 18 — Additional file 18:Supplementary material 18. Fixed-effect meta-analysis of short-term versus long-term cognitivebehavioral therapy for anxiety disorders on anxiety symptoms (sensitivity analysis). [file 12888_2023_4895_MOESM18_ESM.pdf]
