# Supplementary material for: The difference between shorter- versus longer-term psychotherapy for adult mental health disorders: a systematic review with meta-analysis
Source: BMC Psychiatry. 2023 Jun 16;23:438. doi: 10.1186/s12888-023-04895-6 (PMC10273498; doi:10.1186/s12888-023-04895-6)

Supplementary material 19. Fixed-effect meta-analysis of short-term versus long-term psychodynamic psychotherapy for mood- and anxiety disorders on level of functioning (sensitivity analysis)

	Short-term psychodynamic			Long-term psychodynamic				Std. Mean Difference
**Study or Subgroup**	**Mean**	**SD**	**Total**	**Mean**	**SD**	**Total**	**Weight**	**IV, Fixed, 95% CI**
Knekt et al. 2008	1.88	0.55	83	1.72	0.62	107	55.3%	0.27 [-0.02, 0.56]
Lorentzen et al. 2013	-67.8	11.7	71	-68.1	14.2	79	44.7%	0.02 [-0.30, 0.34]
**Total (95% CI)**			**154**			**186**	**100.0%**	**0.16 [-0.05, 0.37]**

Heterogeneity: Chi² = 1.26, df = 1 (P = 0.26); I² = 21%
Test for overall effect: Z = 1.46 (P = 0.14)

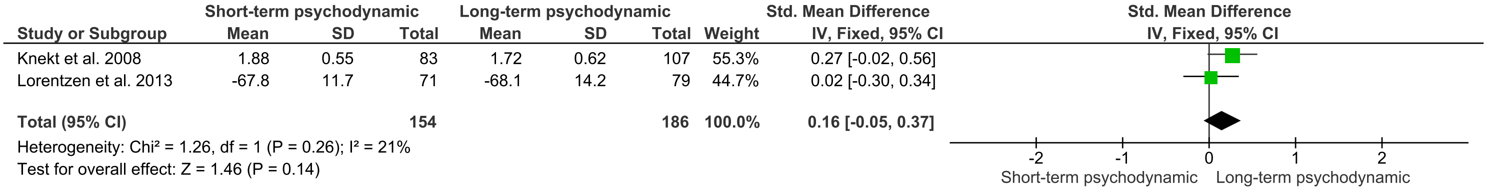

Supplement: Supplementary file 19 — Additional file 19:Supplementary material 19. Fixed-effect meta-analysis of short-term versus long-term psychodynamic psychotherapy for mood- and anxiety disorders on level of functioning (sensitivity analysis). [file 12888_2023_4895_MOESM19_ESM.pdf]
